# Five-Spin Supramolecule for Simulating Quantum Decoherence of Bell States

**DOI:** 10.1021/jacs.2c06384

**Published:** 2022-08-25

**Authors:** Selena J. Lockyer, Alessandro Chiesa, Adam Brookfield, Grigore A. Timco, George F. S. Whitehead, Eric J. L. McInnes, Stefano Carretta, Richard E. P. Winpenny

**Affiliations:** † Department of Chemistry and Photon Science Institute, The University of Manchester, Oxford Road, Manchester M13 9PL, U.K.; ‡ Dipartimento di Scienze Matematiche, Fisiche e Informatiche, Università di Parma, I-43124 Parma, Italy; § INFN−Sezione di Milano-Bicocca, Gruppo Collegato di Parma, I-43124 Parma, Italy; ∥ UdR Parma, INSTM, I-43124 Parma, Italy

## Abstract

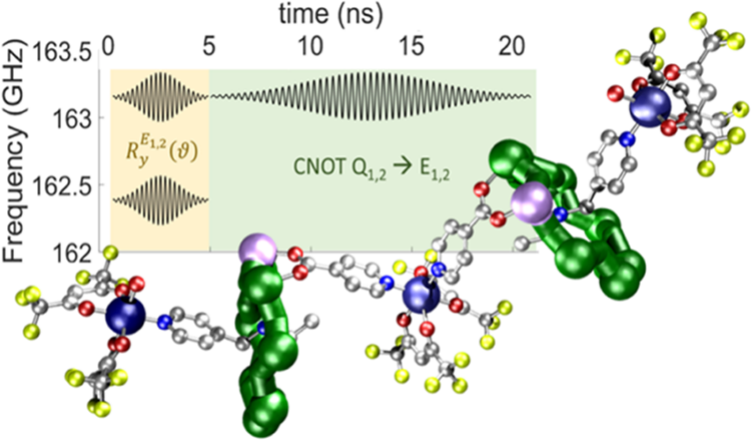

We report a supramolecule that contains five spins of two different types and with, crucially, two different and predictable interaction energies between the spins. The supramolecule is characterized, and the interaction energies are demonstrated by electron paramagnetic resonance (EPR) spectroscopy. Based on the measured parameters, we propose experiments that would allow this designed supramolecule to be used to simulate quantum decoherence in maximally entangled Bell states that could be used in quantum teleportation.

## Introduction

Molecular electron spins could play a key role in the development of coherent nanotechnologies^1^ and in the design of platforms to encode and process quantum information.^2−7^ These molecules can be organized on surfaces^8^ or integrated with superconducting resonators,^9^ the leading technology for solid-state processors. In addition, they can be coherently addressed by both magnetic^10^ and electric field^11^ pulses.

The crucial advantage of molecular electron spins as qubits is the ease with which they can be linked to form more complex spin clusters,^12−16^ targeted for specific quantum information schemes. For instance, the synthesis of supramolecular spin trimers allows one to encode a pair of qubits with a switchable effective qubit–qubit coupling,^17^ a crucial step to implement general quantum computing algorithms in a scalable architecture. Moreover, quantum error correction^18−20^ and quantum simulation^21^ schemes can be proposed based on molecular systems. A question remains whether this advantage compensates for disadvantages such as relatively short coherence times^22−26^ and challenges around addressing the spins.

To realize the advantage, three key steps are needed: first, an ability to make and characterize complex spin clusters that retain the identity of the original qubits; second, a proposed and simulated algorithm that could be performed with the multispin system that would be impossible with a simpler system; and third, the experiment needs to be performed. Here, we report taking the first two steps in a five-spin supramolecule and the simulation of the use of this supramolecular complex as a quantum simulator of the effect of decoherence on two qubits prepared in a maximally entangled state.

Understanding the role of decoherence on the dynamics of a quantum system is of utmost importance both to shed light on fundamental phenomena (such as photosynthetic processes, thermalization, phase transitions) and to design more efficient quantum computing platforms. Indeed, decoherence represents the most important source of errors on any quantum computing hardware. Entanglement is the quintessential quantum phenomenon and a crucial resource for quantum information processing. Hence, by destroying entanglement, decoherence leads to devastating errors in quantum applications. However, simulating the effect of decoherence is very hard because it originates from the interaction of a relatively small subset of qubits with a huge number of environmental degrees of freedom. The resulting dissipative dynamics on the system qubits can be computed along different lines^27−34^ based in general on adding to the system qubits with additional qubits modeling a (weak) coupling to the environment. Here, we show that such a quantum simulation can be performed on a 5-qubit supramolecule with tailored interactions.

**Figure 1:**
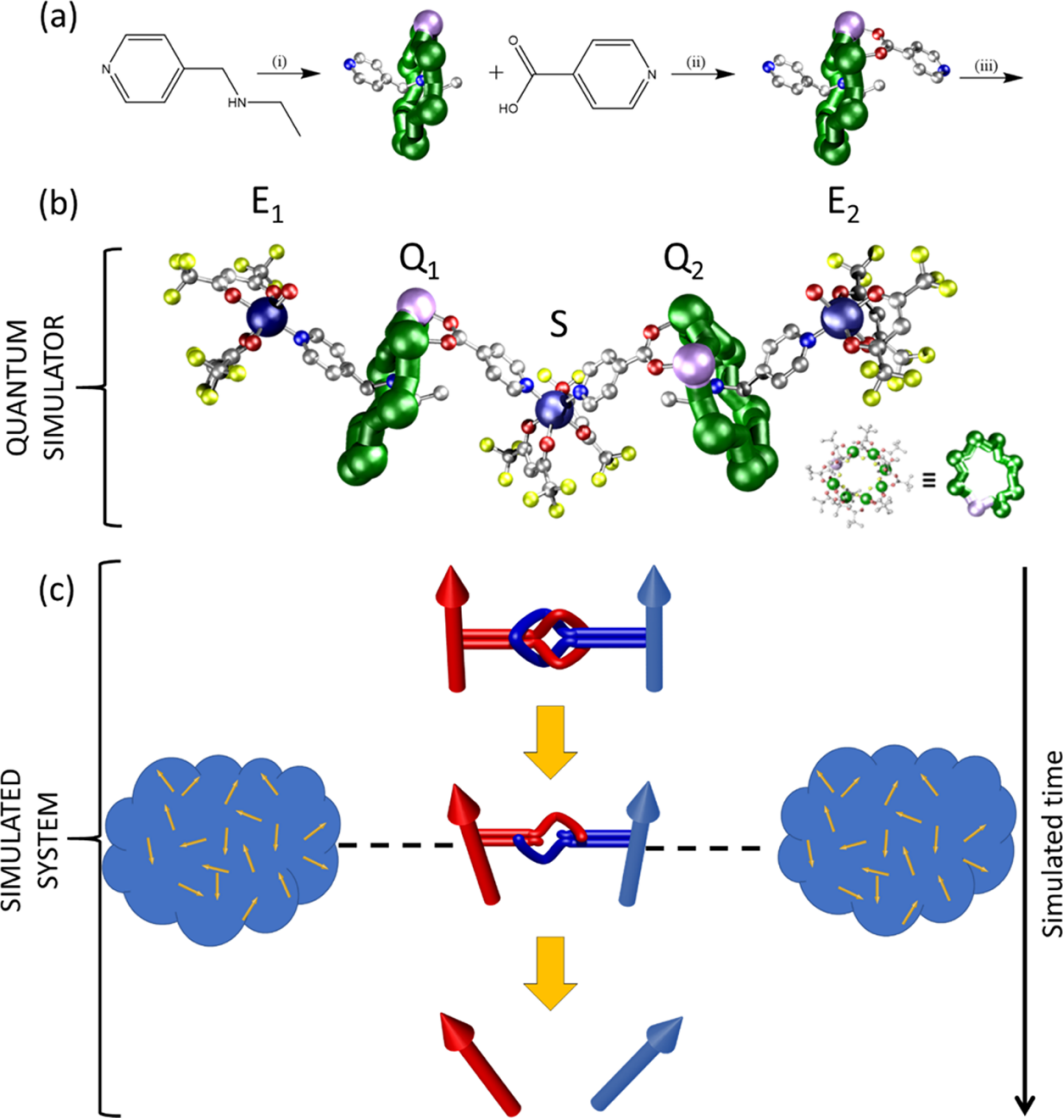
Synthesis and structure of the five-qubit quantum simulator. (a) Scheme for syntheses of **2** and **3**. (i) CrF_3_, xs HO_2_C*^t^*Bu, nickel carbonate, 160° C, 24 h. (ii) *n*PrOH, 24 h. (iii) [Cu(hfac)_2_(H_2_O)_2_], THF/toluene. (b) Structure of **3** in the crystal; the letters S, Q_1_, Q_2_, E_1_, and E_2_ identify the role of the units in the quantum simulator. The colored balls correspond to different atom types: Cr (green), Cu (dark blue), Ni (lilac), O (red), N (blue), F (yellow), carbon (silver). Hydrogens and pivalate groups are omitted for clarity. Inset: full structure of the {Cr_7_Ni} ring. (c) Scheme of the simulated time evolution of the entangled state of Q_1_–Q_2_. The simulated interaction (dashed lines) between the system (Q_1_, Q_2_, big arrows) and the external or the environment (E_1_ and E_2_, clouds) qubits induces decoherence on the system, thus breaking entanglement (sketched as a cord) in a controlled way as the time of the quantum simulation goes on.

The spin cluster we report (Figure 1) is based on linking together {Cr_7_Ni} rings that have been long studied as qubits^12,16,17^ as they have a ground state with S = 1/2, with reasonable coherence times.^22^ Here, we link the rings in two distinct ways, producing a supramolecule containing two interaction energies differing by > an order of magnitude. We characterize the system, and we show how to implement a quantum simulation of decoherence, acting on a pair of entangled qubits (Figure 1c). This is done by exploiting the two very different interactions within the molecule: the larger one defines a core unit of two “system” qubits (given by the Cr_7_Ni rings), with a switchable interaction provided by the central Cu (auxiliary qubit exploited as a switch of the effective coupling between Cr_7_Ni qubits). The smaller interaction is used to simulate the weak coupling of the system with the “environment”.

As a case study, we prepare the system in a Bell state, i.e., a maximally entangled two-qubit state used, for instance, in quantum teleportation (QT). Indeed, the success of QT is based on the entanglement between the qubits shared by the two parties involved in the teleportation. Hence, we compute the failure probability of the QT protocol as a measure of the break of entanglement induced by decoherence.

We perform thorough numerical simulations of the whole algorithm, based on experimental parameters, finding a very good agreement with expected results. This shows that the present supramolecular system could form the building block of a quantum simulator, capable of mimicking the dynamics of an open quantum system.

## Results and Discussion

### Synthesis and Structural Characterization

We have previously linked {Cr_7_Ni} rings in two ways. The first is by introducing a binding group in the periphery of the ring—a “covalent” link—by replacing an inert pivalate group with a binding *iso*-nicotinate group.^12^ Second, we linked them through a cationic thread terminated with a pyridyl that sits at the center of the ring—a “supramolecular” link.^35^ The covalent link can be bound to Cu^II^ centers, producing a magnetic exchange interaction between the {Cr_7_Ni} ring and the Cu^II^ as large as 0.5 cm^–1^. The supramolecular link when bound to Cu^II^ centers typically leads to interaction energies orders of magnitude smaller.^35^ Here, we introduce both interactions in one supramolecule.

A {Cr_7_Ni} pseudo-rotaxane was prepared by forming a {Cr_7_Ni} around a commercially available secondary amine to produce [(pyCH_2_NH_2_Et)][Cr_7_NiF_8_(O_2_C*^t^*Bu)_16_] **1** (where py = pyridyl, C_5_H_4_N) as previously reported (see Figure 1a).^35^ The pseudo-rotaxane **1** was then further functionalized by substitution of a carboxylate localized at the Ni(II) ion, producing [(pyCH_2_NH_2_Et)][Cr_7_NiF_8_(O_2_C*^t^*Bu)_15_(O_2_C-py)] **2**. The structure of **2** shows that the ring now contains two potential binding groups: a nitrogen within an *iso*-nicotinate ligand and a pyridyl that terminates the thread of the pseudo-rotaxane (Figure S1). Compound **2** is the vital component in our five-spin ensemble as when it is mixed in THF with stoichiometric amounts of [Cu(hfac)_2_(H_2_O)_2_] (hfac = 1,1,1,6,6,6-hexafluoroacetylacetonate), it produces {[Cu(hfac)_2_(H_2_O)-**2**]_2_-[Cu(hfac)_2_]} **3** in good yield. The structure of **3** is shown in Figure 1b.

Compound **3** crystallizes from THF and toluene. The central copper(II) site sits on a twofold rotation axis and is six-coordinate-bound to four O-donors from hfac^–^ ligands and to two *cis* N-donors from *iso-*nicotinates. This will be the switch (S) in our quantum simulator. The second copper(II) sites are also six-coordinate, bound to four O-donors from hfac^–^ ligands, one N-donor from a thread, and one H_2_O ligand, which is *cis*- to the N-donor. These will be the external sites in the quantum simulator. The two distinct copper sites are bridged by **2** with the *iso*-nicotinate group bound to the central S Cu^II^ and the thread N-donor bound to the external E Cu^II^ sites. The overall compound contains five individual spin s = 1/2 units. We have seen both types of ring···Cu interactions previously, in two-spin (supramolecular Cu···ring via thread)^35^ or three-spin (covalent ring···Cu···ring via *iso*-nicotinates)^12^ systems, but never together in the same molecule.

The Jahn–Teller axis for the S copper site is easily distinguished and lies along the only O···Cu···O vector; these Cu–O bonds are 2.24(1) Å. The other Cu···O bonds and the Cu–N bonds are 2.00(2) and 2.01(2) Å, respectively. The Jahn–Teller axis is not as obvious for the E copper sites; the Cu–N bond is 2.00(1) Å long and the Cu–O bond *trans* to it is 1.97(1) Å long. The other four Cu–O bonds range from 2.06(1) to 2.10(1) Å. Therefore, there appears to be a small Jahn–Teller compression. The shorter Cu1···Cu2 contact is 15.21(1) Å, and the distance between the two terminal Cu2 sites is 28.05(1) Å. The Cu···Cu···Cu angle is 134°.

The bond lengths within the {Cr_7_Ni} rings, which are our qubits Q, are unremarkable. The ring centroid–ring centroid distance is 16.41(1) Å. The angle between the mean planes of the rings is 53°; therefore, the inter-ring metal···metal distances range from 13.76(1) to 21.09(1) Å. We have previously shown that the *g*_*z*_ direction is perpendicular to the mean plane of the {Cr_7_Ni} ring.^36^

### Electron Paramagnetic Resonance Spectroscopy

Continuous-wave (CW) Q-band (ca. 34 GHz) electron paramagnetic resonance (EPR) spectroscopy measurements were performed on **3** at 5 K for powder samples and for a 3 mM 1:1 CH_2_Cl_2_/toluene solution.

**Figure 2:**
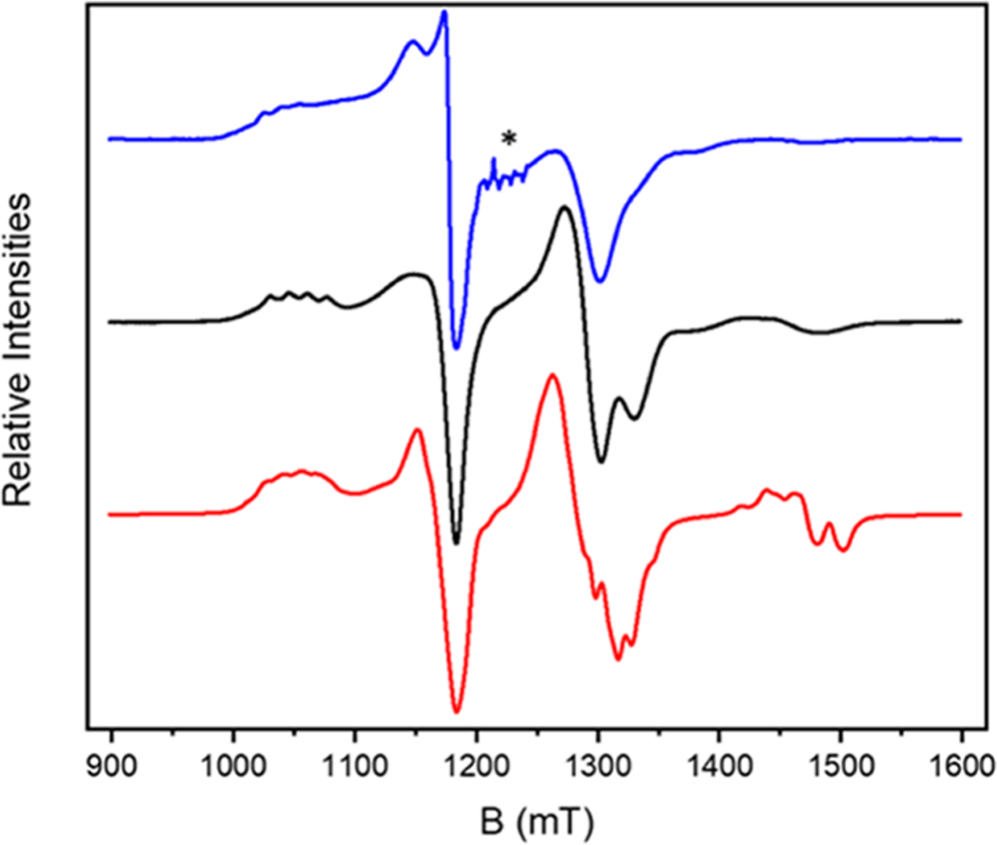
5 K Q-band (34.068575 GHz) EPR spectra of **3**. Experimental solution (blue) and powder spectra (black) and a calculated spectrum (red) using previously reported parameters as stated in the text and a line width of 12 mT. * indicates a Mn impurity in the tube.

The EPR spectra of **3** are similar in solution and as a powder (Figure 2). Multiple features are observed at 5 K ranging from 1000 to 1600 mT. As the structure of **3** is built from components we have previously seen separately,^12,35^ we calculated a spectrum^37^ using fixed parameters that we have previously reported for the various components (Figure 2) and the spin Hamiltonian below;
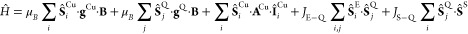
where the superscripts E, S, and Q label the external and internal Cu ions and the rings, respectively. There are no free variables other than the line width. These parameters (listed as *x, y, z*, where *x*, *y*, and *z* refer to the local **g**-frames of the components) are as follows: *g*_Q_: 1.785, 1.785, and 1.750; *g*_Cu_: 2.065, 2.085, and 2.325; *A*_Cu_: 20, 20, and 500 MHz; *J*_S-Q_ = 9,174 MHz; *J*_E-Q_ = 780 MHz. The local *g*_*z*_ axes of the components are fixed in the orientations defined by the structures (see above); hence, *g_z_* of the S Cu is perpendicular to those of the Q rings. The *g*_Cu_ and *A*_Cu_ are used for both the switch and external Cu^II^ sites.

This calculation, with two fixed and isotropic unique exchange interactions, is in remarkably good agreement with the experimental spectrum of **3**. The stronger coupling (9174 MHz, 0.306 cm^–1^) is due to the covalent interaction through the *iso*-nicotinate.^12,38^ The weaker coupling (780 MHz, 0.026 cm^–1^) is the supramolecular interaction via the thread. The value used is that found for **1** bound to [Cu(hfac)_2_].^35^ We estimate the uncertainties in these *J* values as 4 and 15%, respectively, from test calculations varying each in turn (Figures S4 and S5). By control of the chemistry, we can predictably vary the interaction energy between our qubits by over an order of magnitude within the same supramolecule. Moreover, we have shown that the exchange coupling constants are transferable between parent fragments and more complex supramolecular systems.

The agreement with previous measurements on related complexes also indicates that the identity of the qubits is retained in the five-spin complex. The measured phase memories for **3** are 1.00, 1.08, and 0.76 μs at 1045, 1169, and 1291 mT; the first two field positions correspond to Cu^II^ resonances and the last to the {Cr_7_Ni} ring (Figure 2),^35^ in line with parameters measured for the individual units. These values were then used in the quantum simulation discussed below.

### Quantum Simulation of Decoherence

The five-spin system **3** has remarkable features for quantum information applications: (i) the identity of the qubits is retained after creating the supramolecular cluster; and (ii) the very different interaction strengths of the Cr_7_Ni rings with the central and external Cu^II^ ions. Property (i) is a fundamental requirement to define any quantum register containing several qubits, whose reciprocal interaction must be switched on and off to implement general algorithms. Property (ii) allows **3** to be used to perform a quantum simulation of decoherence on Bell states.

In the proposed setup, Cr_7_Ni rings are system qubits (labeled as Q_1_ and Q_2_ in Figure 1b). The central copper is the switch (S) of the effective ring–ring interaction, and it is exploited to prepare the Bell state of the system. The two external Cu^II^ sites (E_1_, E_2_) are used to induce decoherence in a controlled way, i.e., simulating the coupling between the quantum system and the environment. The significant difference between the values of *J*_S-Q_ and *J*_E-Q_ is an important resource for the proposed simulation. The large value of *J*_S-Q_ enables fast implementation of conditional dynamics of Q_1_–Q_2_ and, hence, fast preparation of the initial state. On the other hand, the much smaller *J*_E-Q_ keeps the state of the two system qubits Q_1_ and Q_2_ factorized from that of E_1_ and E_2_, thus enabling decoherence to be induced in a controlled way.

**Figure 3:**
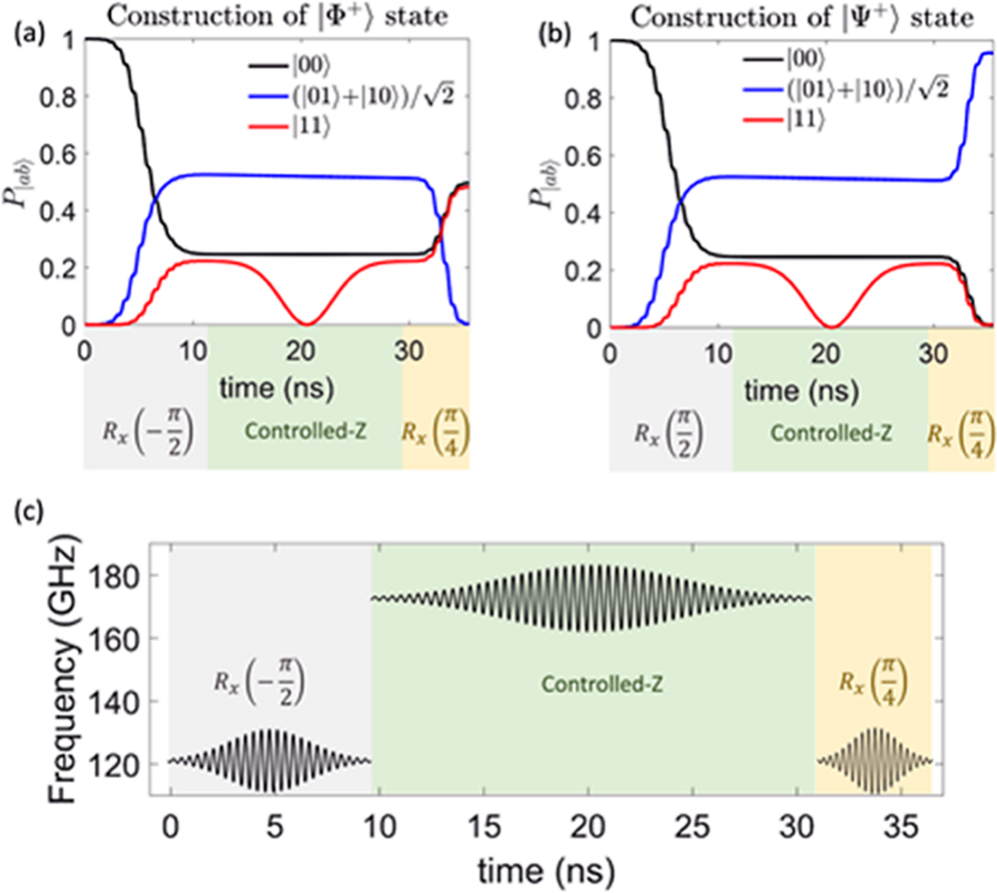
Construction of maximally entangled Bell states on the system qubits. (a, b) Time evolution of the probability of finding each of the |Q_1_Q_2_⟩ eigenstates during the implementation of the pulse sequence (reported in the bottom) needed to prepare Bell states |Φ^+^⟩ (a) or |Ψ^+^⟩ (b), including the effect of finite experimental phase memory times for the different subunits, namely, 0.76 μs for Cr_7_Ni and ∼1 μs for Cu. (c) Employed pulse sequence, consisting of pulses addressing either Q_1,2_ (∼120 GHz) for initial and final rotations or *S* (∼170 GHz) to implement the controlled-Z two-qubit gate on |Q_1_Q_2_⟩.

We illustrate our scheme to implement the quantum simulation of decoherence on **3**, proceeding in two steps. First, we prepare the two qubits Q_1_–Q_2_ in one of the maximally entangled two-qubit states known as Bell states^39^ (Figure 3a,b). We consider |Φ^+^⟩ = (|00⟩ + |11⟩)/√2 and |Ψ^+^⟩ = (|01⟩ + |10⟩)/√2, where 0 and 1 correspond to ↓ and ↑ states of the two Cr_7_Ni rings in a significant applied field (the other two Bells states are equivalent). To keep factorized eigenstates and limit residual coupling when the switch is off, we consider a static field of 5 T parallel to *z* (in a global reference frame parallel to Cu principal axes), such that (*g*_S_^*zz*^ – *g*_Q_^*zz*^)μ*_B_B* ≫ *J*.^17,40^ The significant value of *J*_S-Q_ makes the excitation of S dependent on the state of both Q_1_ and Q_2_. Starting with the whole register in the ground state |↓↓↓↓↓⟩, we build Bell states by combining symmetric single-qubit rotations on Q_1_ and Q_2_ (obtained by pulses resonant with the Cr_7_Ni transitions) with a two-qubit controlled-*Z* gate (see pulse sequence in Figure 3c and Supporting Information). The latter is obtained by a 2π resonant transition of S, conditioned on the state of both Q_1_ and Q_2_ being ↓. By tuning the rotation angle, we can prepare states |Φ^+^⟩ or |Ψ^+^⟩ by choice (Figure 3), with remarkable fidelity, including the finite phase memory time on the different subunits in the simulation. Despite being symmetric on the two qubits, the controlled-*Z* gate (which adds a π phase only to the |11⟩ component of the *Q*_1_-*Q*_2_ wave function) implements conditional dynamics and it is able to generate an entangled pair.

As a second step, we simulate the effect of decoherence by inducing an evolution of the E_1_ and E_2_ spins (the external Cu spins, modeling the environment) depending on the state of Q_1_ and Q_2_ (the rings). As detailed in the SI, this can be done by directly exploiting the Q_1,2_–E_1,2_ coupling *J*_E-Q_, which makes all transitions of the Q*_i_*–E*_i_* pairs distinguishable.^14,41^ In particular, we implement a *R*_*y*_(ϑ) on E_1,2_, followed by a CNOT gate between Q_1,2_ (control) and E_1,2_ (target). By finally measuring only the state of the system qubits,^42^ we effectively mimic decoherence on {Q_1_,Q_2_} in a controlled way. In particular, the angle  is mapped to the decay of coherences in the system density matrix by sin ϑ = e^–*t*/*T*_2_^, where *t* is the simulated time in units of the dephasing time *T*_2_ of the simulated system (see SI). Hence, by tuning ϑ in the *R*_*y*_ rotation of E_1,2_, we simulate the amount of time during which the system is subject to dephasing (see Figure 1c).

**Figure 4:**
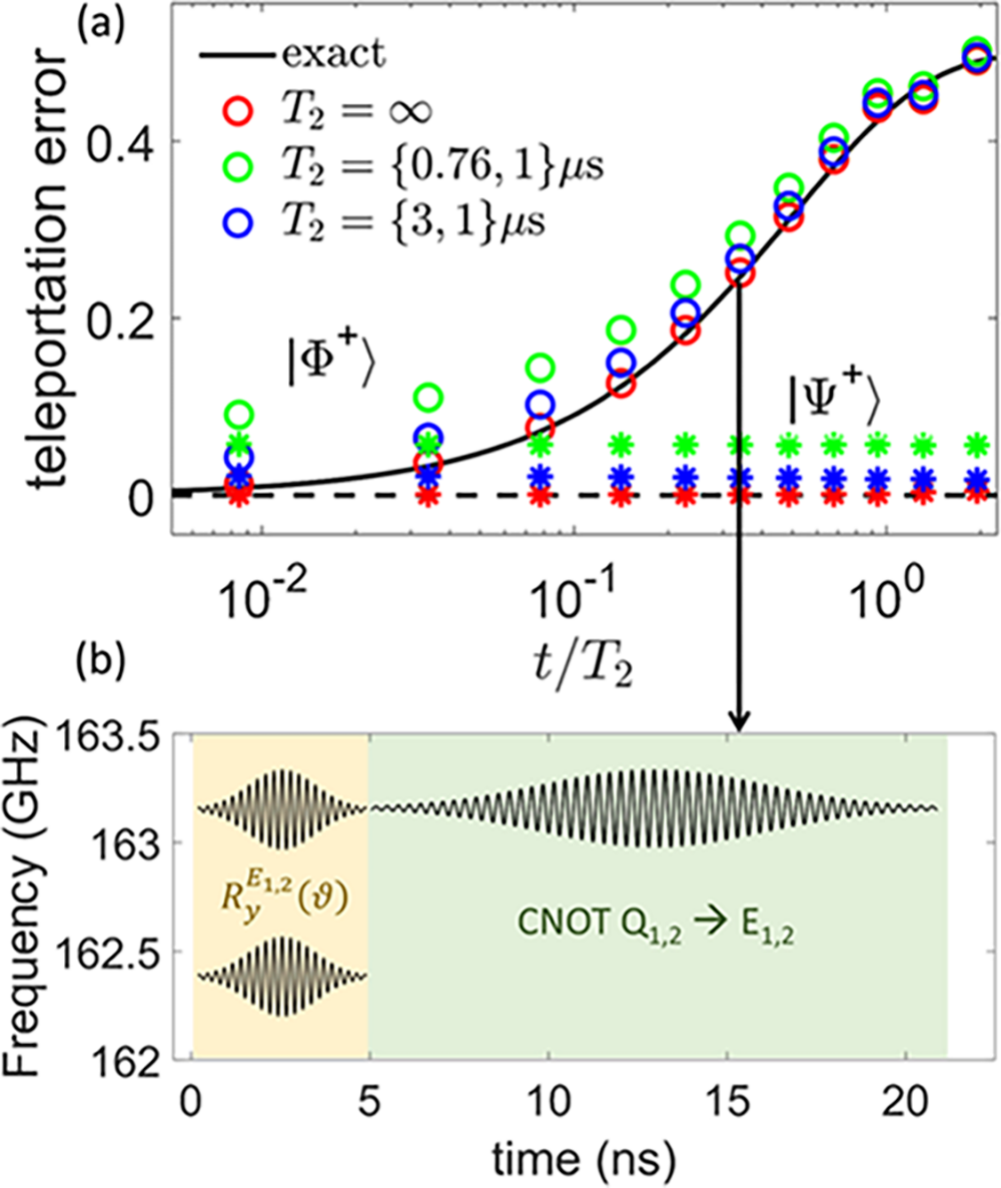
Quantum simulation of decoherence on the Bell state, quantified by the error in a quantum teleportation protocol (a), as a function of the waiting time before teleportation (in units of the simulated dephasing time *T*_2_). The continuous (dashed) black line represents the exact expected result for a pair of system qubits initialized in |Φ^+^⟩ (|Ψ^+^⟩). Colored circles (stars) are the corresponding simulations without including the finite phase memory times of the subunits (red), with the experimental ones (green) for Cr_7_Ni and Cu and with reasonable values, which can be obtained by deuteration (blue). (b) Pulse sequence implementing the simulation, in which only the external (E_1,2_) spins are addressed, with the frequency depending on the state of the neighboring Q_1,2_. Rotations (yellow background) require two slightly different frequencies to be performed independent of the state of Q_1,2_, while the CNOT between Q_1,2_ (control) and E_1,2_ (target) is obtained with a single pulse.

As a prototypical application of our scheme, we consider a quantum teleportation (QT) experiment, in which an entangled Bell pair shared between two parties is exploited to transfer a quantum state between each other.^39^ The failure probability of the algorithm is directly related to the break of entanglement induced by decoherence. Hence, we use the error after QT^43^ as a measure of the effect of decoherence on the initial entangled system state. Results are shown in Figure 4a, as a function of the waiting time between preparation and actual teleportation (in units of the dephasing time *T*_2_). The pulse sequence needed to compute each point is shown in Figure 4b, where only E_1,2_ spins are addressed (either for rotations or as target of the CNOT). The expected behavior (black lines) is different for the two initial states |Φ^+^⟩ and |Ψ^+^⟩: in the former, the two-quantum coherence between |00⟩ and |11⟩ components is subject to a decay rate twice that of a single spin 1/2, with an error on QT increasing in time from 0 to 0.5. Conversely, |Ψ^+^⟩ (characterized by only a zero-quantum coherence between |01⟩ and |10⟩) is immune from dephasing, and hence, QT is error-free in the presence of a symmetric interaction of the two spins with the environment.

This opposite behavior is well reproduced by our simulations (symbols), even in the presence of the experimentally measured phase memory times, of about 760 ns on Cr_7_Ni units (green). We note, however, that phase memory times of about 3 μs could be obtained by simply removing hydrogen nuclei via deuteration,^22^ without further chemical optimization. By making such a reasonable assumption, the simulated (blue) points are practically superimposed on those obtained without including qubit dephasing in the simulations (red), thus demonstrating that **3** performs very well as a quantum simulator of decoherence.

## Conclusions

We have made and characterized a complex array of five qubits suited to perform the quantum simulation of a pair of qubits (encoded in the Cr_7_Ni rings), subject to decoherence. If these qubits are exploited in a quantum teleportation protocol, the effect of decoherence is to induce an error on the teleported state due to loss of entanglement within the original Bell state of the pair of qubits. We obtain such a simulation by exploiting the central Cu as a switch of the interaction between the rings, thus preparing a Bell state at will by proper pulses. The external Cu ions are instead used to mimic the effect of the environment. Results of our simulations, including measured values of all of the parameters, show a very good agreement with the expected behavior, demonstrating that **3** can actually work as a quantum simulator of the dynamics of an open quantum system.

The proposed scheme can be implemented on an ensemble of molecules, and the final state of only the system Cr_7_Ni spins can be read out by spectroscopic techniques. This, in turn, requires full state tomography of the two-qubit density matrix (see SI). The experimental realization requires microwave pulses resonant with transitions of all of the subunits. Here, due to the relatively large value of *J*_S-Q_, we considered a magnetic field of 5 T to keep the state of the two rings factorized from that of the switch, thus avoiding unwanted residual evolution. As shown in Figures 3c and 4b, this implies microwave pulses of frequencies approximately 120 and 170/160 GHz to address Q and S/E, respectively. Nevertheless, it is important to note that a proof-of-principle experiment can already be performed with not perfectly factorized states, using **3** and a magnetic field of about 3.5 T, corresponding to W-band EPR frequencies.

In general, since factorization of the eigenstates scales as *J*/*B*, a small reduction of *J*_S-Q_ would allow a proportional reduction of the static field and hence of the pulse frequencies, keeping the same (almost ideal) conditions used in our simulations. This would require reducing *J*_S-Q_ by a factor 1.5 to move to the W-band. This is chemically achievable.^35^

Another possibility to reduce the static field is to have a slight asymmetry between the two rings because the corresponding difference between Zeeman energies of the rings leads to factorized states. A similar asymmetry (e.g., a rotation of the coordination environment) could also be useful to separately address the two external ions, thus simulating the effect of an asymmetric coupling to the environment.

An experimental realization of the proposed scheme requires using significantly different microwave frequencies. This could be done by employing a multimode resonator; it has been suggested that excitation bandwidths of 4 GHz could be possible.^44^ Alternatively, one could employ an architecture based on superconducting resonators, where frequencies differing by more than a factor of 7 have already been demonstrated.^45^ These devices typically operate in the few tens of GHz range.^46^ This, in turn, would require a reduction of the applied field and hence of the exchange couplings, which is feasible in this chemistry.

The proposed approach is general and allows one to simulate the dynamics of a generic open quantum system (e.g., a linear chain of qubits), subject to other dissipative processes, such as relaxation or the depolarizing channel.^42^ The basic principles are the same as illustrated above for our specific example: given a decomposition of the incoherent evolution into Kraus operators,^39^ these are translated into a linear sum of *d* unitary operators, which are simulated with the addition of a *d*-dimensional ancilla to the system qubits. Finally, by measuring the system state independently from the ancillary one (i.e., summing over all states of the ancillae), we achieve a simulation of the dissipative dynamics of the system.

## Supporting Information Available

Experimental section; ESI mass spectroscopy, crystallography; c.w. EPR data; quantum simulations; and construction of Bell states, quantum simulations, and quantum state tomography (PDF)
