# Supplementary material for: Five-Spin Supramolecule for Simulating Quantum Decoherence of Bell States
Source: J Am Chem Soc. 2022 Aug 25;144(35):16086–92. doi: 10.1021/jacs.2c06384 (PMC9460766; doi:10.1021/jacs.2c06384)
Supplement: Supplementary file 1 — ja2c06384_si_001.pdf [file ja2c06384_si_001.pdf]

Supporting Information

A five-spin supramolecule for simulating quantum decoherence of Bell states

Selena J. Lockyer,[1] Alessandro Chiesa,[2,3,4] Adam Brookfield,[1] Grigore A. Timco,[1] George F. S. Whitehead,[1] Eric J. L. McInnes,[1] Stefano Carretta[2,3,4]* and Richard E. P. Winpenny[1]*.

1. Department of Chemistry and Photon Science Institute, The University of Manchester, Oxford Road, Manchester M13 9PL, UK.

2. Università di Parma, Dipartimento di Scienze Matematiche, Fisiche e Informatiche, I-43124 Parma, Italy.

3. INFN–Sezione di Milano-Bicocca, gruppo collegato di Parma, I-43124 Parma, Italy.

4. UdR Parma, INSTM, I-43124 Parma, Italy.

Contents

1. Synthesis S2
2. ESI Mass Spectroscopy S4
3. Crystallography S10
4. EPR Spectroscopy S14
5. Quantum simulation S19
6. References S22

1. Synthesis

General: All starting reagents and materials used were sourced from Sigma-Aldrich and/or Alfa. Unless stated otherwise, all reagents and solvents were used without further purification. The syntheses of the hybrid organic-inorganic rotaxanes were carried out in Erlenmeyer Teflon® FEP flasks supplied by Fisher. Column chromatography was performed using either 40-63 µm silica from Sigma-Aldrich or a Grace Reverelis ® X2 Autocolumn with Grace Reverelis ® NP cartridges. Chemical shifts are reported in parts per million (ppm) from low to high frequency and referenced to the residual solvent resonance. ESI mass spectrometry and microanalysis were carried out by the services at The University of Manchester.

1.1 Synthesis of $[Cr_7NiF_8(O_2C^tBu)_{16}(pyCH_2NH_2Et)]$ **1**.

Pivalic acid (25 g, 245 mmol), $CrF_3.4H_2O$ (3 g, 16 mmol), $2NiCO_3.3Ni(OH)_2.4H_2O$ (0.35g, 0.6 mmol) and N-(4-pyridylmethyl)ethylamine (0.35 mL, 2.5 mmol) were added to a Teflon flask and heated to 160 °C for 24 hours. The mixture was left to cool to room temperature, acetonitrile (35 mL) was added, then stirred for 30 minutes. The mixture was filtered and washed with acetonitrile (150 mL). Column chromatography (10:1 toluene:ethyl acetate) produced two fractions, the second one containing the product. Solvents were removed to produce a green powder. Yield 1.48 g 25% ESI MS m/z (relative intensity) 2403$[M+H]^+$, 2425 $[M+Na]^+$, 2441$[M+K]^+$.

1.2 Synthesis of $[Cr_7NiF_8(O_2C^tBu)_{15}(O_2C\text{-}py)(pyCH_2NH_2Et)]$ **2**.

Isonicotinic Acid (0.37 g, 3 mmol), was added to a solution of **1** (3.5 g, 1.5 mmol) in 1-propanol and refluxed (115 °C) in an open condenser for 24 hours. The solution was left to cool to RT before the solvent was reduced by half, then acetonitrile (100 mL) was added with stirring for 20 minutes. The precipitate was filtered with washing from acetonitrile (100 mL). Column chromatography (1.5:1 DCM:ethyl acetate) produced three fractions, the third one containing the product. Solvents were removed to produce a green powder. Yield: 0.9 g 26% ESI MS /z (relative intensity) 2352$[M+H]^+$, 2374 $[M+Na]^+$,

2390[M+K]$^+$. Elemental analysis %: calc. for $C_{89}H_{152}Cr_7F_8N_3NiO_{32}$: Cr 15.48, Ni 2.50, C 45.47, H 6.52, N 1.79; found: Cr 15.70, Ni 2.73, C 43.96, H 6.75, N 1.58.

Crystallisation was achieved by dissolving **2** (0.25 g) in warm acetone (15 mL, 40 °C) and left to cool to RT with slow evaporation. Green crystals suitable for single crystal X-ray diffraction formed slowly over 48 hours. The crystals were separated by filtration and washed with acetone. Yield: 0.05 g (20%).

1.3 Synthesis of {[$Cr_7NiF_8(O_2C^tBu)_{15}(O_2C$-py)(py$CH_2NH_2$Et)][Cu(hfac)$_2$] [Cu(hfac)$_2H_2O$)]$_2$} **3**.

To a warm solution (~40 °C) of **2** (0.1 g, 0.042 mmol) in THF (15 mL), copper(**II**) hexafluoroacetylacetonate hydrate (0.03 g, 0.063 mmol) was added and the solution stirred for 10 minutes, filtered and left to cool. Then toluene (5 mL) was added and the solvent was allowed to slowly evaporate. Dark green crystals suitable for single crystal X-ray diffraction formed slowly over 96 hours. The crystals were separated by filtration and washed with THF. Yield (based on **2**): 0.035 g (27%). Elemental analysis %: calc. for $C_{208}H_{314}Cr_{14}Cu_3F_{52}N_6Ni_2O_{78}$: Cr 11.80, Ni 1.90, Cu 3.09, C 40.49, H 5.13, N 1.36; found: Cr 10.57, Ni 1.76, Cu 3.16, C 41.82, H 5.15, N 1.51.

To check the g_{Cu} and $J_{S\text{-}Q}$ parameters used to simulate the EPR spectra for **3** were correct a further molecule was synthesised without the pyridyl thread.

[$Cr_7NiF_8(O_2C^tBu)_{15}(O_2C$-py)($^nPr_2NH_2$)] **4** was synthesised as described in reference 12.

1.4 Synthesis of {[$Cr_7NiF_8(O_2C^tBu)_{15}(O_2C$-py)($^nPr_2NH_2$)][Cu(hfac)$_2$]} **5**.

To a warm solution (~40 °C) of **4** (0.1 g, 0.043 mmol) in THF (15 mL), copper(**II**) hexafluoroacetylacetonate hydrate (0.01 g, 0.022 mmol) was added and the solution stirred for 10 minutes, filtered and left to cool. Then toluene (5 mL) was added and the solvent was allowed to slowly evaporate. Dark green crystals suitable for single crystal X-ray diffraction formed slowly over 72 hours. The crystals were separated by filtration and washed with THF. Yield: 0.028 g (24%). Elemental analysis %: calc. for

$C_{184}H_{312}Cr_{14}CuF_{28}N_4Ni_2O_{68}$: Cr 14.25, Ni 2.30, Cu 1.24, C 43.25, H 6.16, N 1.10; found: Cr 13.45, Ni 2.29, Cu 1.22, C 45.36, H 6.34, N 1.69.

A related compound has been reported previously in reference S1 but there the nicotinate ligands were *trans* about the central $Cu(hfac)_2$ unit rather than *cis*.

2. ESI mass spectroscopy experimental and calculated spectra

2.1 ESI mass spectroscopy spectra for **1**.

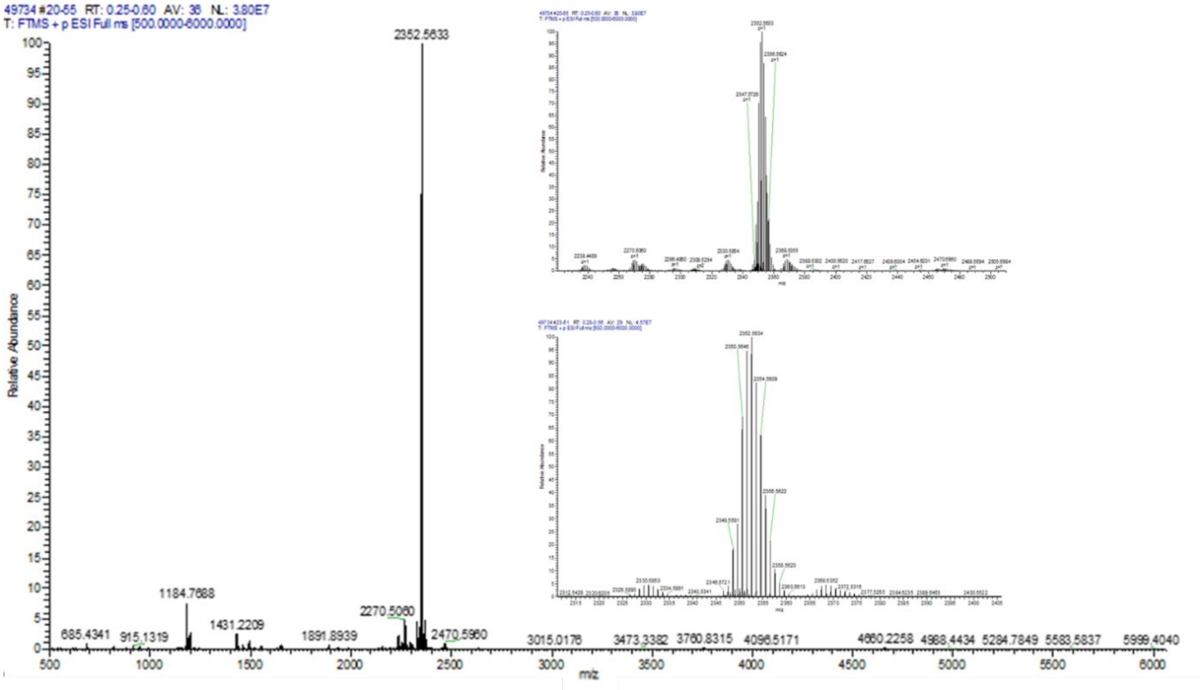

Figure S1: Experimental ESI mass spectroscopy for **1**. Inserts: Enlarged section for 2220 - 2250 m/z, top. Zoomed to show details for +H, +Na and +K peaks, bottom.

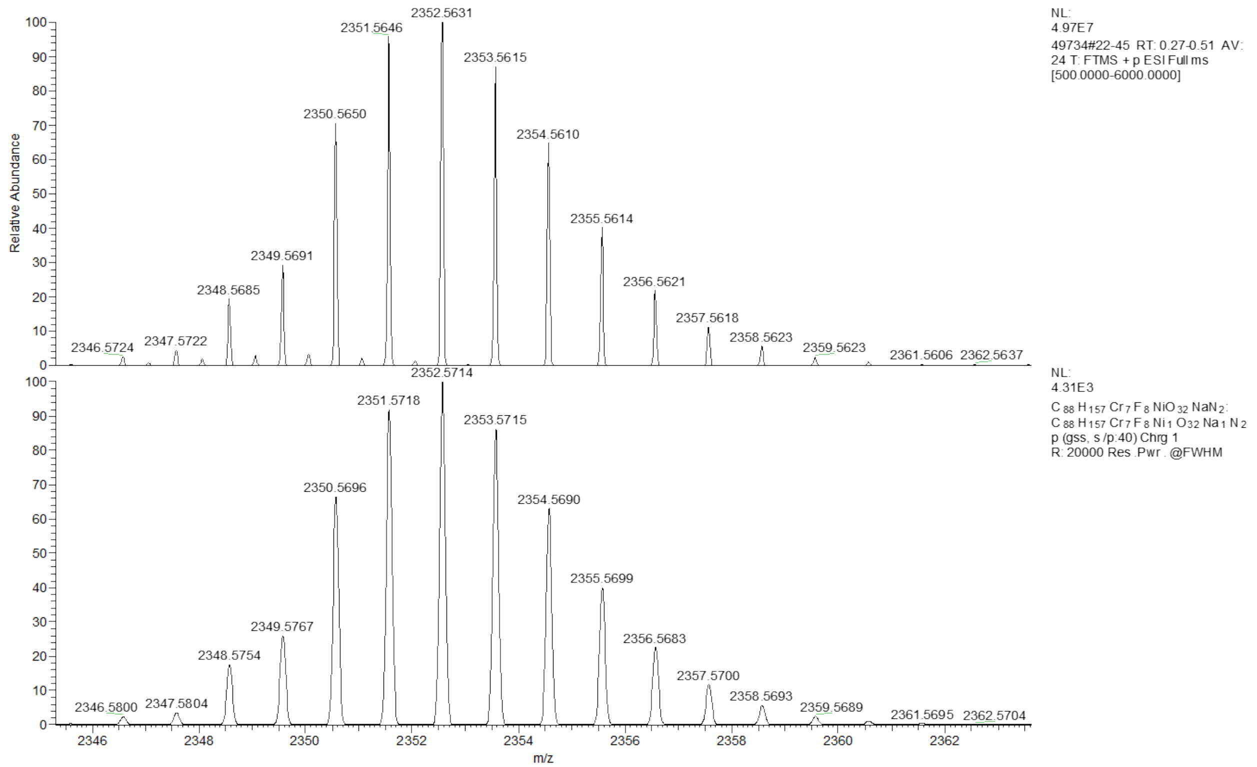

Figure S2: Experimental and calculated ESI mass spectroscopy for +Na peaks for **1**. Top and bottom, respectively.

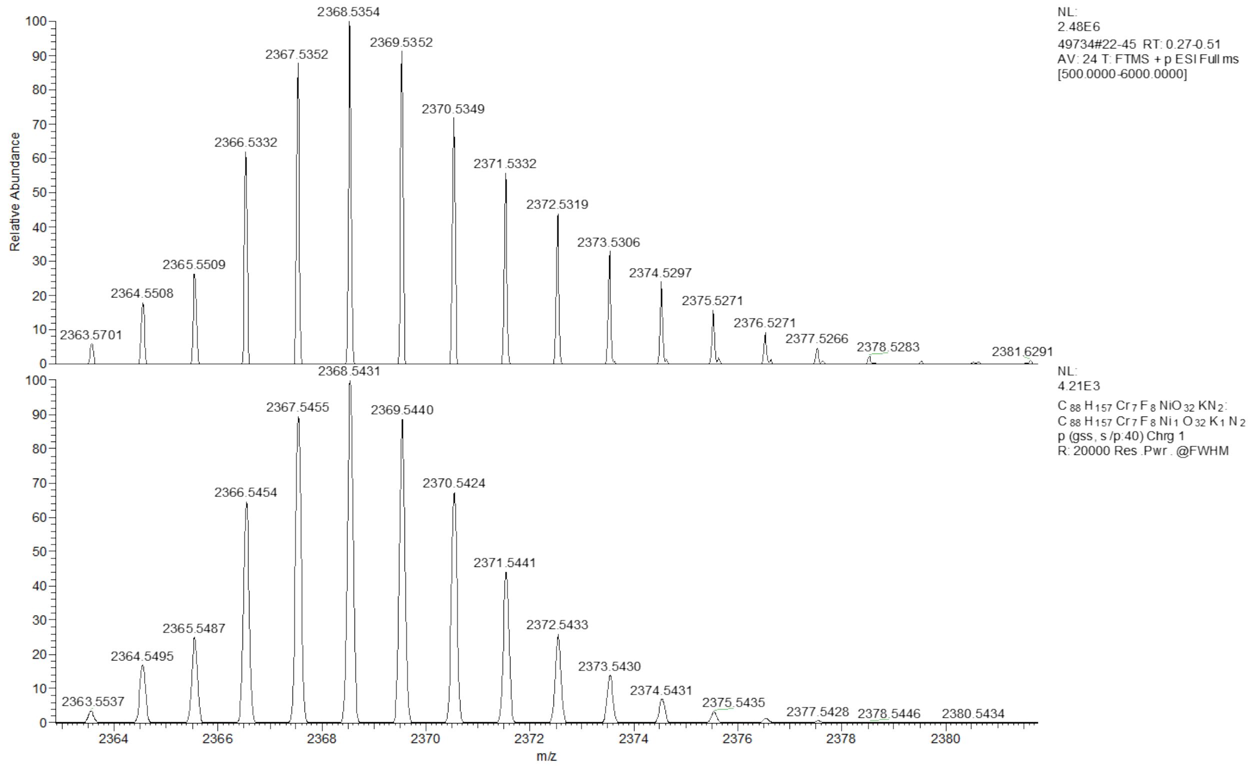

Figure S3: Experimental and calculated ESI mass spectroscopy for +K peaks for **1**. Top and bottom, respectively.

2.2 ESI mass spectroscopy spectra for **2**.

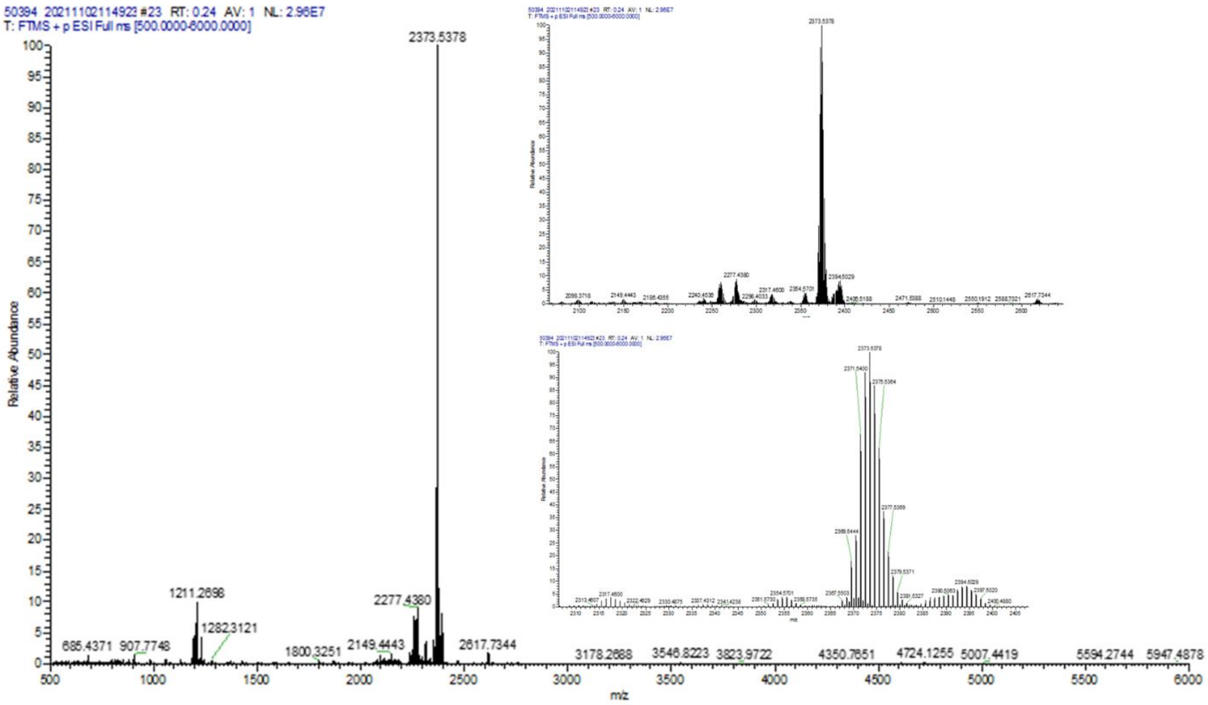

Figure S4: Experimental ESI mass spectroscopy for **2**. Inserts: Enlarged section for 2050 - 2750 m/z, top. Zoomed to show details for +H, +Na and +K peaks, bottom.

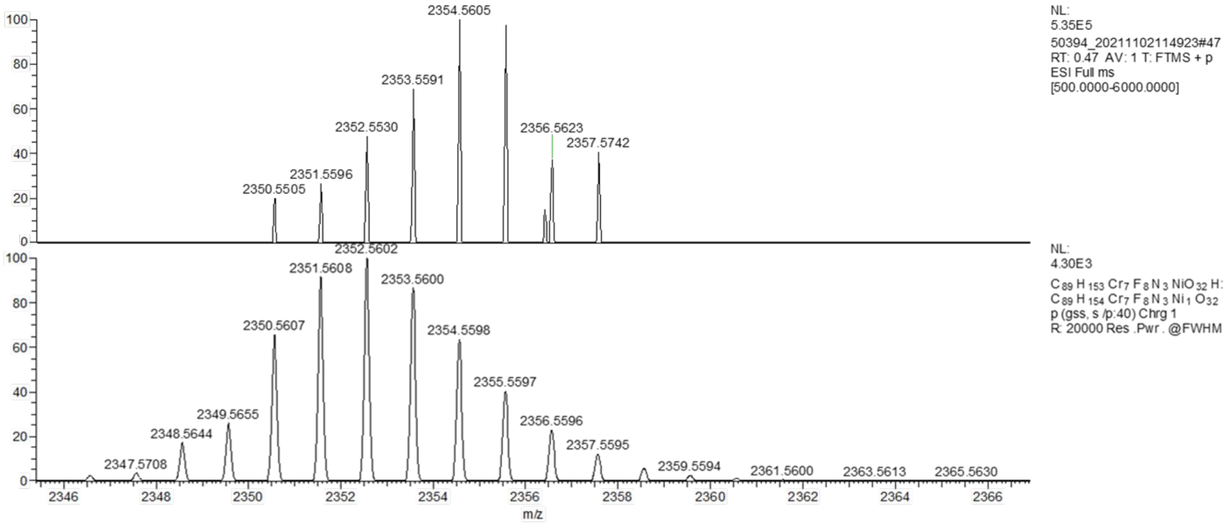

Figure S5: Experimental and calculated ESI mass spectroscopy for +H peaks for **2**. Top and bottom, respectively.

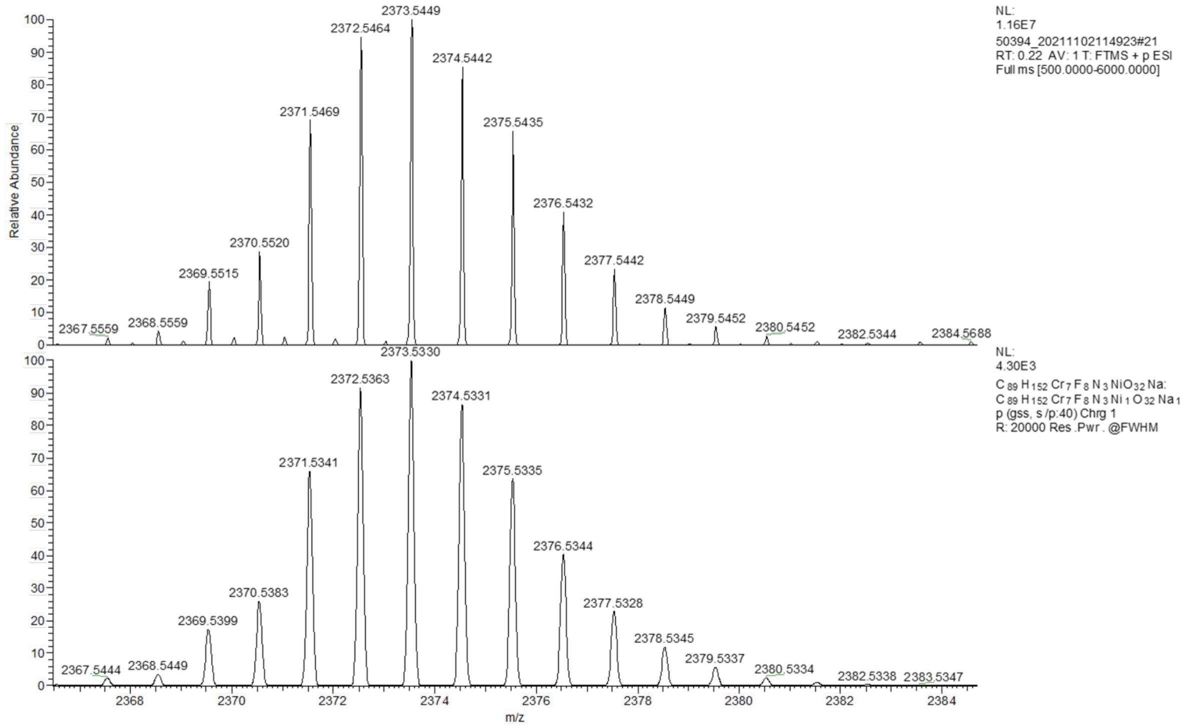

Figure S6: Experimental and calculated ESI mass spectroscopy for +Na peaks for **2**. Top and bottom, respectively.

2.3 ESI mass spectroscopy spectra for **4**.

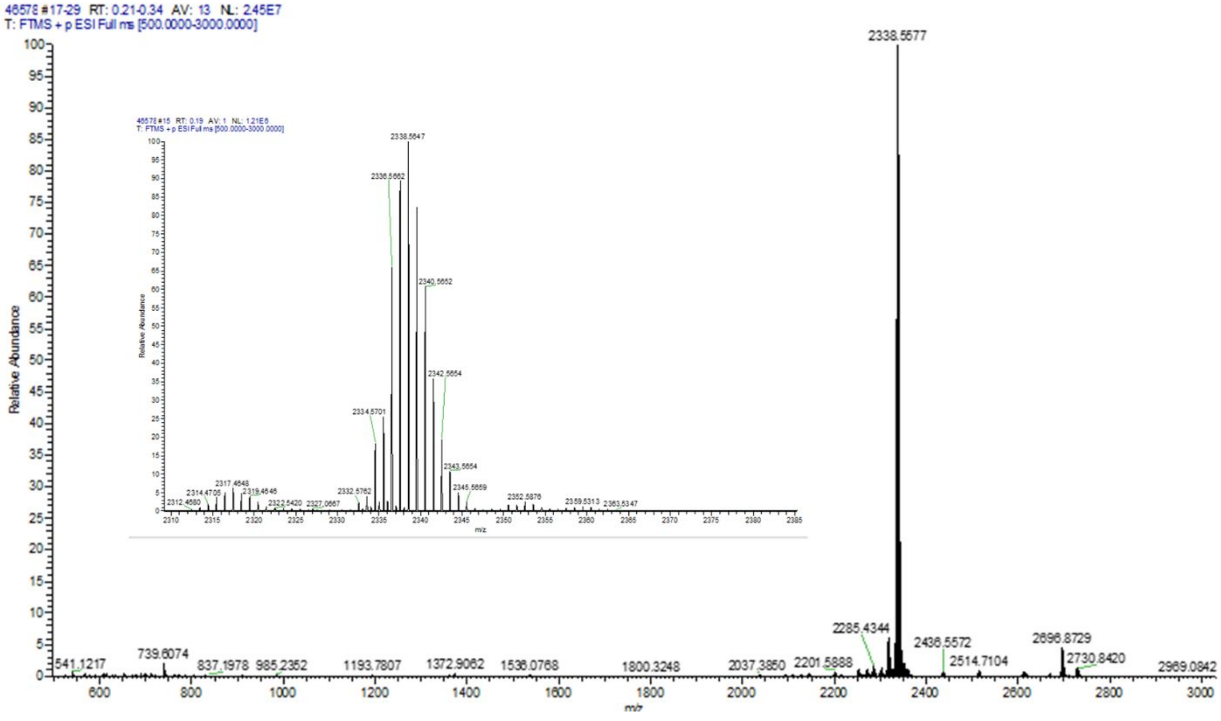

Figure S7: Experimental ESI mass spectroscopy for **4**. Insert, zoomed to show details for +H and +Na peaks.

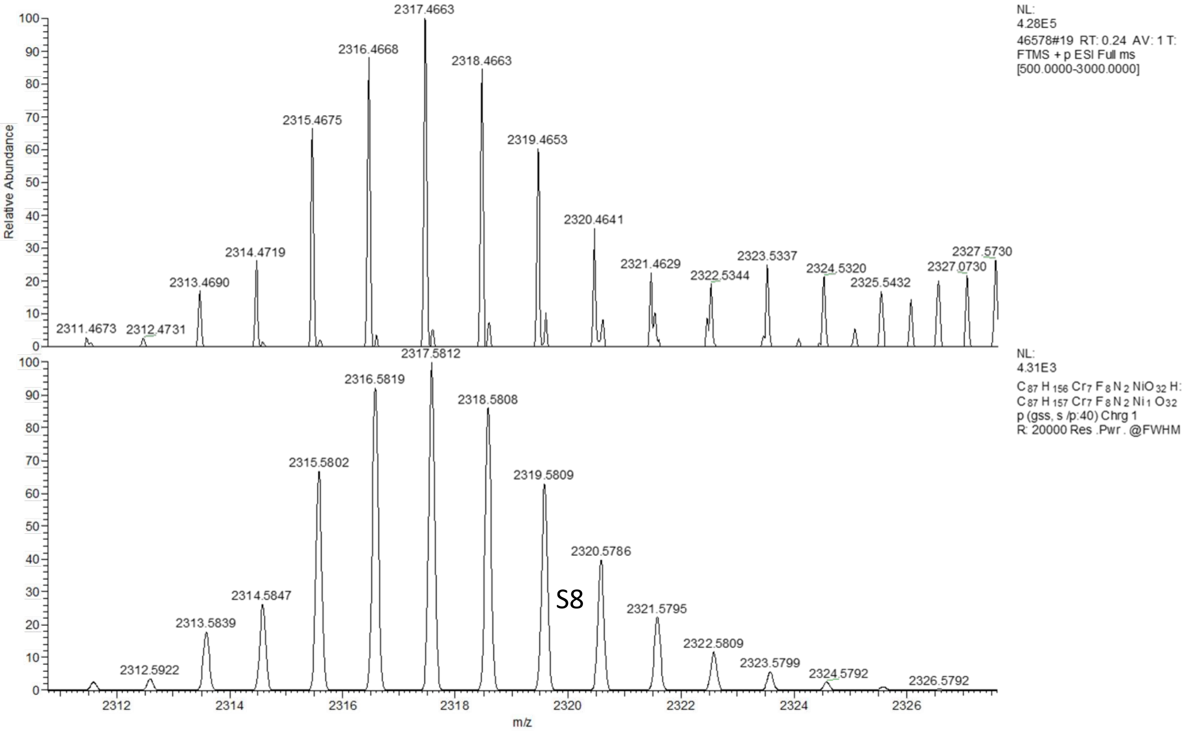

Figure S8: Experimental and calculated ESI mass spectroscopy for +H peaks for **4**. Top and bottom, respectively.

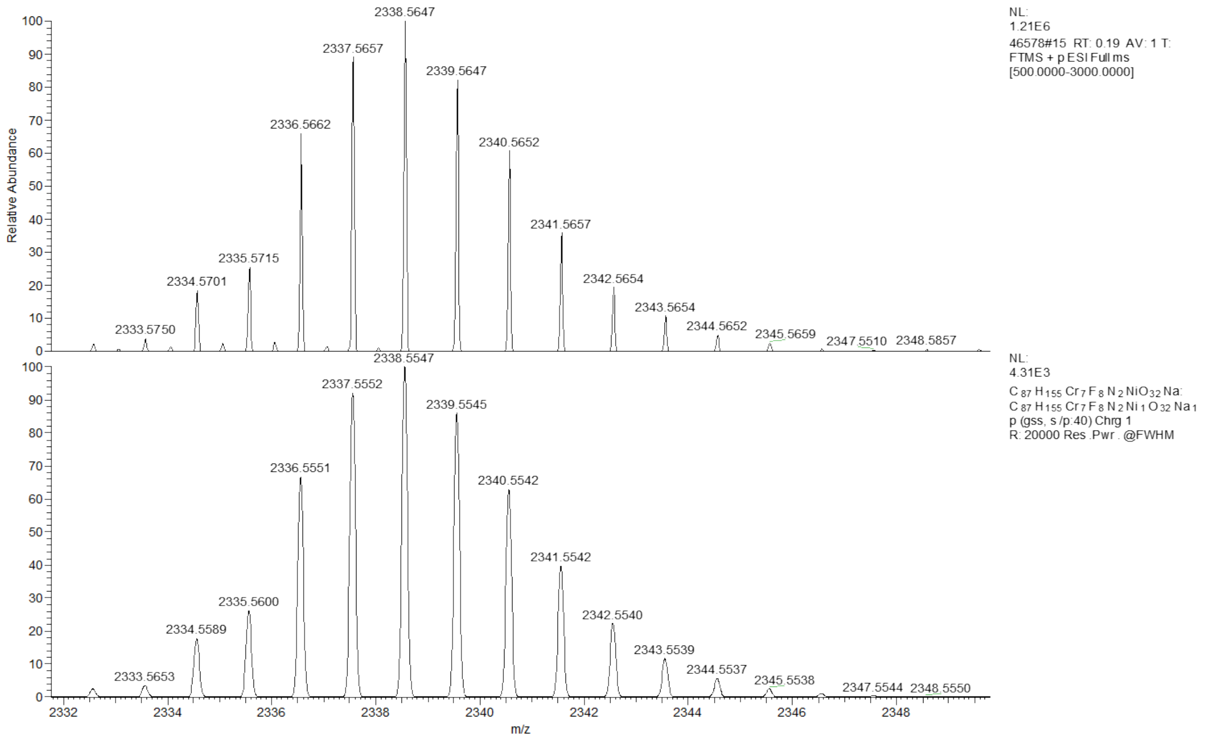

Figure S9: Experimental and calculated ESI mass spectroscopy for +Na peaks for **4**. Top and bottom, respectively.

3. Crystallography

Data Collection

X-Ray data for compounds **2**, **3** and **5** were collected at a temperature of 100 K using a Rigaku FR-X with Cu-Kα (**2** and **3**), and Mo-Kα (**5**) radiation equipped with a Hypix 6000HE detector, equipped with an Oxford Cryosystems nitrogen flow gas system. Data was measured using CrysAlisPro suite of programs.

Crystal structure determinations and refinements. X-Ray data were processed and reduced using CrysAlisPro suite of programs. Absorption correction was performed using empirical methods (SCALE3 ABSPACK) based upon symmetry-equivalent reflections combined with measurements at different azimuthal angles.[S2] The crystal structure was

solved and refined against all F^2 values using the SHELXL and Olex 2 suite of programmes.[S3]

All atoms in crystal structures were refined anisotropically with the exception of the hydrogen atoms, which were placed in the calculated idealized positions for all crystal structures. The pivalate ligands, and threads in crystal structures were disordered and modelled over two positions, using structural same distance (SADI) and distance fix (DFIX) Shelxl restraints commands. The atomic displacement parameters (adp) of the ligands have been restrained using similar Ueq and rigid bond (SIMU) and Similar Ueq (SIMU) restraints.

CCDC 2171151 - 2171153 contains the supplementary crystallographic data for this paper. These data can be obtained free of charge *via* www.ccdc.cam.ac.uk/conts/retrieving.html (or from the Cambridge Crystallographic Data Centre, 12 Union Road, Cambridge CB21EZ, UK; fax: (+44)1223-336-033; or deposit@ccdc.cam.ac.uk).

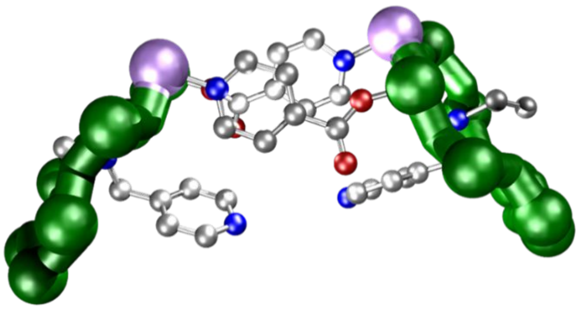

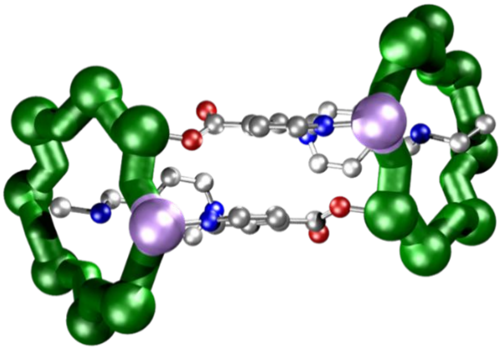

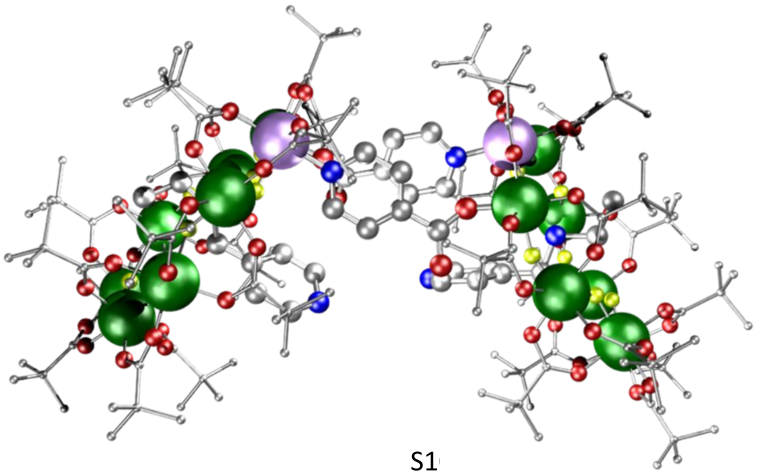

Figure S10: Structure of **2** in the crystal. **2** crystallises as a dimer via the iso-nicotinic pyridyl groups binding to the opposing Ni(II) site. Colour scheme as per manuscript. Top: Hydrogen and pivalic acid groups omitted for clarity. Left: side view, Right: plane view. Bottom: Including pivalic acid groups.

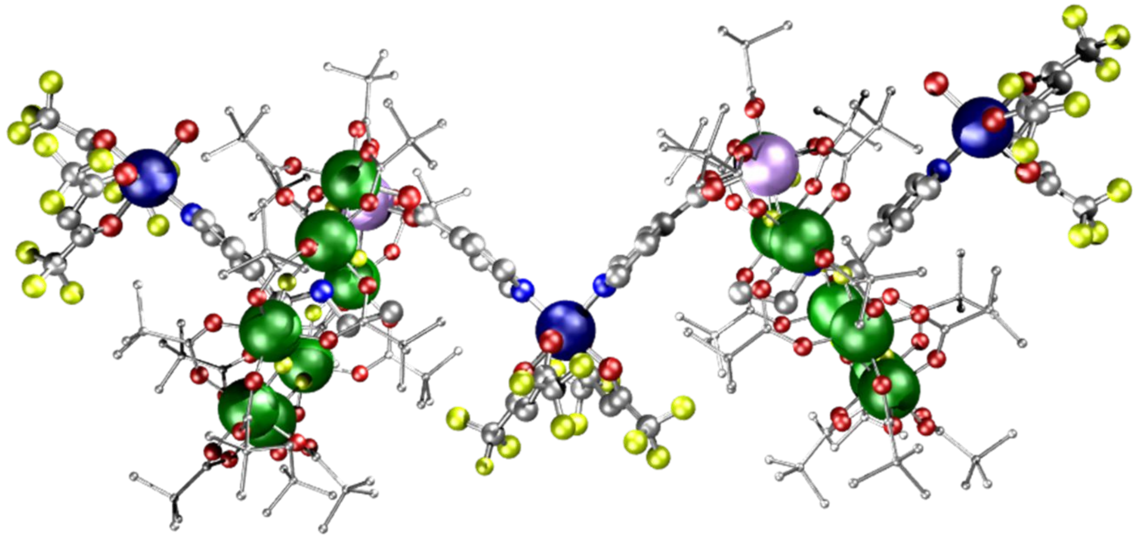

Figure S11: Figure of **3**. Top: Hydrogen and pivalic acid groups omitted for clarity. Colour scheme as per manuscript. Bottom: Including pivalic acid group.

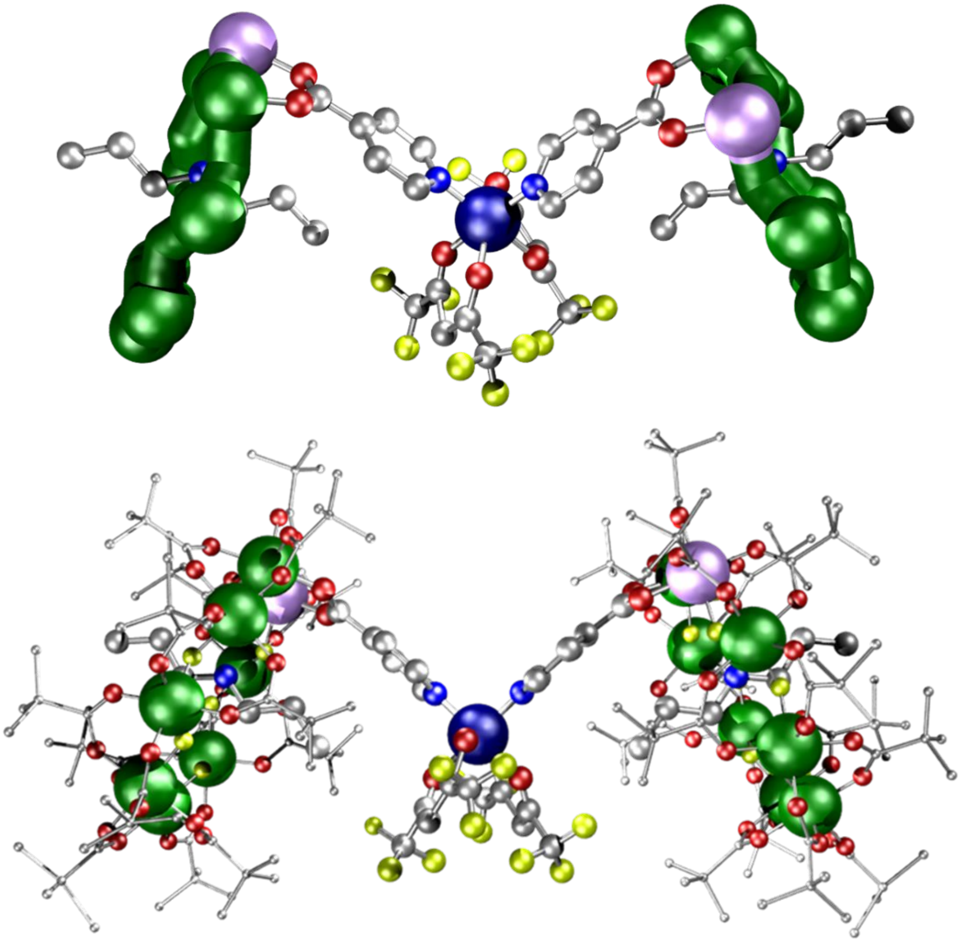

Figure S12: Figure of **5**. Top: Hydrogen and pivalic acid groups omitted for clarity. Colour scheme as per manuscript. Bottom: Including pivalic acid group.

Crystallographic Tables:

Identification code	**2**	**3**	**5**
Empirical formula	$C_{221.5}Cr_{14}F_{16}H_{393}N_6Ni_2O_{79.5}$	$C_{250}Cr_{14}Cu_3F_{52}H_{366}N_6Ni_2O_{80}$	$C_{184}H_{312}Cr_{14}CuF_{28}N_4Ni_2O_{68}$
Formula weight	5561.82	6759.51	5109.32
Temperature/K	99.97(10)	99.99(10)	99.99(11)
Crystal system	monoclinic	orthorhombic	orthorhombic
Space group	$P2_1/n$	Pccn	Pccn
a/Å	26.4469(9)	16.6636(4)	16.9568(2)
b/Å	34.9712(8)	62.905(4)	54.7272(7)
c/Å	33.2883(12)	31.0214(11)	30.4219(4)
α/°	90	90	90
β/°	109.893(4)	90	90
γ/°	90	90	90

Volume/Å^3	28950.6(17)	32517(2)	28231.5(6)
Z	4	4	4
ρ_{calc}g/cm^3	1.276	1.381	1.323
μ/mm^{-1}	4.989	4.897	0.807
F(000)	11744.0	13980.0	11764.0
Crystal size/mm^3	0.156 × 0.048 × 0.027	0.106 × 0.064 × 0.008	0.381 × 0.209 × 0.058
Radiation	Cu Kα (λ = 1.54184)	Cu Kα (λ = 1.54184)	Cu Kα (λ = 0.71073)
2Θ range for data collection/°	3.788 to 119.152	5.486 to 91.866	2.976 to 61.706
Reflections collected	126066	68743	214447
Independent reflections	42326<br>R_{int} = 0.0990<br>R_{sigma} = 0.1181	13754<br>R_{int} = 0.1339<br>R_{sigma} = 0.1203	35008<br>R_{int} = 0.0552<br>R_{sigma} = 0.0482
Data/restraints/parameters	42326/12801/2810	13754/4924/1681	35008/4866/1607
Goodness-of-fit on F^2	1.006	1.021	1.092
Final R indexes [I>=2σ (I)]	R_1 = 0.0742,<br>wR_2 = 0.1827	R_1 = 0.0965,<br>wR_2 = 0.2287	R_1 = 0.0871,<br>wR_2 = 0.2065
Final R indexes [all data]	R_1 = 0.1425,<br>wR_2 = 0.2141	R_1 = 0.1610,<br>wR_2 = 0.2621	R_1 = 0.1105,<br>wR_2 = 0.2177
Largest diff. peak/hole / e Å^{-3}	0.58/-0.55	0.60/-0.41	0.98/-0.84

4. EPR Spectroscopy

Continuous wave Q-band (~34 GHz) EPR spectra were recorded with a *Bruker EMXPlus* spectrometer equipped with a *Bruker ER5106QT flexline* resonator. Cryogenic temperatures were achieved using a *Bruker Stringer* closed cycle helium cryocooler mated to an *Oxford Instruments CF935 cryostat*. Temperature holding and regulation was controlled using an *Oxford Instruments MercuryITC*. The continuous wave data were collected on polycrystalline powders at 5 K (unless otherwise stated). All continuous wave spectra were field corrected using a *Bruker* 'Strong Pitch' standard (*g* = 2.0028) and all powder samples were checked for any polycrystalline nature, by measuring multiple random rotations.

Pulsed Q-band (~34 GHz) EPR data was collected on a Bruker ELEXSYS 580 FT spectrometer. The pulse data was collected from dry and degassed toluene solutions at 3 K (unless otherwise stated) using a Cryogenic cryogen free variable temperature cryostat incorporating a closed helium circuit.

Spectral simulations were performed using the EasySpin 5.2.30 software[S4] with a spin-Hamilton for **3** incorporating the individual **g**-matrices, the Cu hyperfine interaction, and two independent isotropic exchange interactions:

$$\hat{\mathrm{H}} = \mu_{\mathrm{B}}\sum_{i}\hat{\mathbf{S}}_{i}^{Cu}\cdot\mathbf{g}^{Cu}\cdot\mathbf{B} + \mu_{\mathrm{B}}\sum_{i}\hat{\mathbf{S}}_{\mathrm{i}}^{Q}\cdot\mathbf{g}^{Q}\cdot\mathbf{B} + \sum_{\mathrm{i}}\hat{\mathbf{S}}_{i}^{Cu}\cdot\mathbf{A}^{Cu}\cdot\hat{\mathbf{I}}_{i}^{Cu} + J_{E-Q}\sum_{\mathrm{i}}\hat{\mathbf{S}}_{i}^{E}\cdot\hat{\mathbf{S}}_{j}^{Q} + J_{S-Q}\sum_{\mathrm{i}}\hat{\mathbf{S}}_{i}^{Q}\cdot\hat{\mathbf{S}}^{S}$$

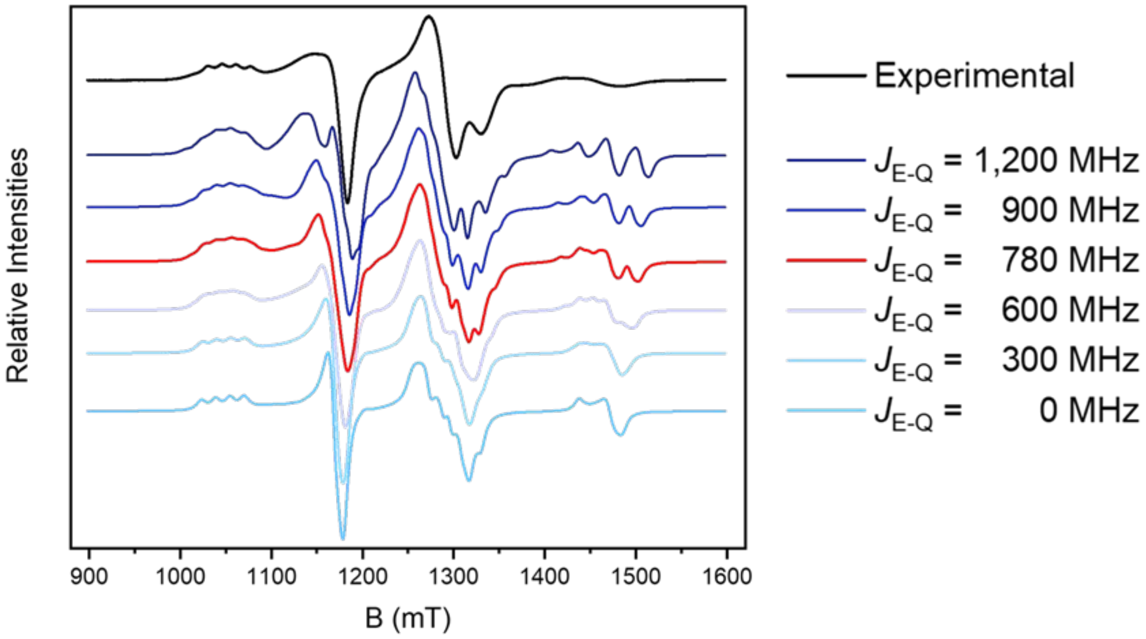

Figure S13: Simulation for **3** varying J_{E-Q}, with fixed J_{S-Q} (using a *J* Hamiltonian). Black line is the experimental 5 K powder. The red line is using J_{E-Q} from reference 35.

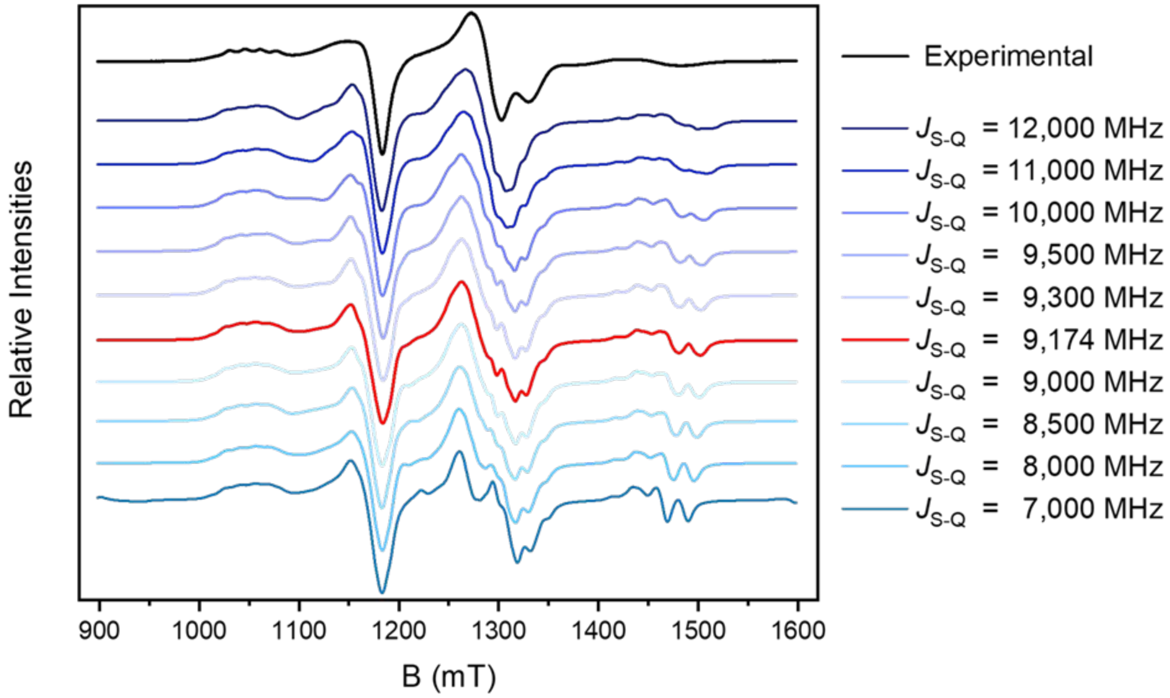

Figure S14: Simulation for **3** varying J_{S-Q}, with fixed J_{E-Q} (using a *J* Hamiltonian). Black line is the experimental 5 K powder. The red line uses J_{S-Q} from reference 12.

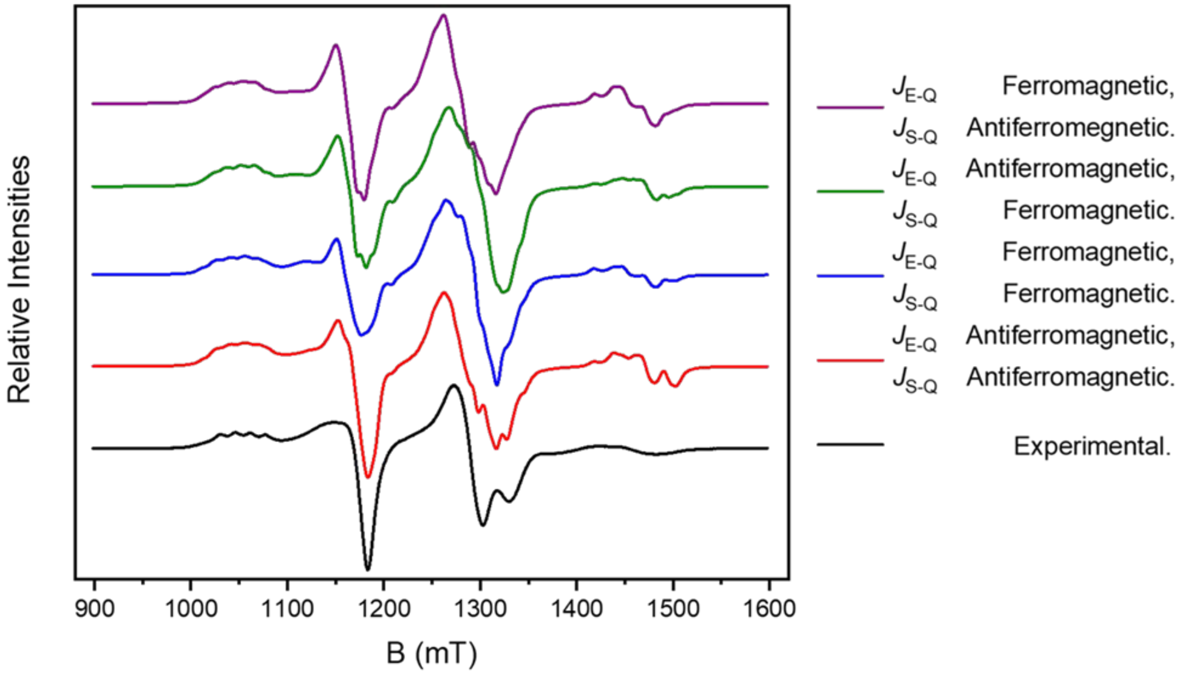

Figure S15: Simulation for **3** with ferro- and anti-ferromagnetic exchange couplings with magnitudes fixed at values from references 12 and 35.

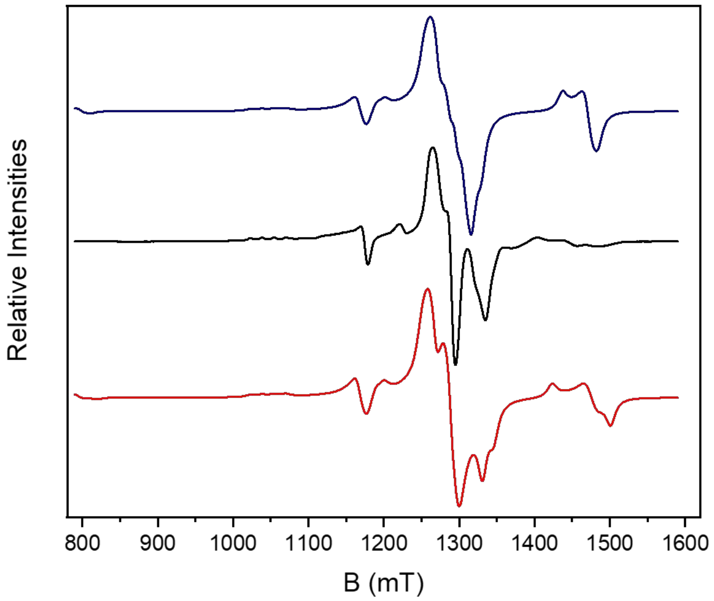

Figure S16: CW Q-Band EPR experimental for **5** as a powder at 5K (black line) and a calculation (blue line) using the same parameters as for **3** but omitting the two terminal Cu(II) ions. A simulation of the experimental spectrum is shown in red and uses the identical parameters except g_Q = 1.798, 1.790, 1.723. This is the only difference from the blue line where g_Q = 1.785, 1.785, 1.750. Experimental frequency: 34.052705 GHz.

The spin-Hamilton for **5** incorporates the individual **g**-matrices, the Cu hyperfine interaction, and an independent isotropic exchange interactions:

$$\hat{\mathrm{H}} = \mu_{\mathrm{B}}\hat{\mathrm{S}}^{Cu} \cdot \mathbf{g}^{Cu} \cdot \mathbf{B} + \mu_{\mathrm{B}}\hat{\mathrm{S}}^{Cr7Ni} \cdot \mathbf{g}^{Cr7Ni} \cdot \mathbf{B} + \hat{\mathrm{S}}^{Cu} \cdot \mathbf{A}^{Cu} \cdot \hat{\mathrm{I}}^{Cu} + J_{S-Q}\sum \hat{\mathrm{S}}^{Cu} \cdot \hat{\mathrm{S}}^{Cr7Ni}$$

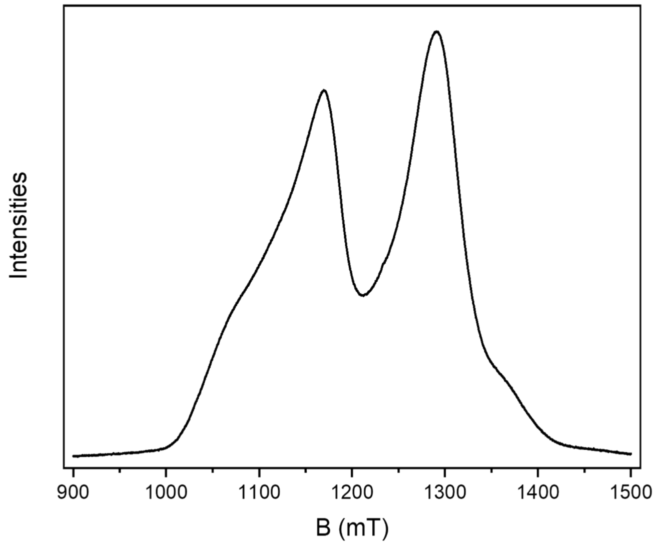

Figure S17: Q-Band (ca. 34 GHz) echo detected field sweep spectra of **3** at 3 K, in 0.1 mM solution in dry and degassed toluene. The two peaks maxima echo intensity corresponding at B_0 = 1169 mT and B_0 = 1291 mT, for the left and right peaks respectively.

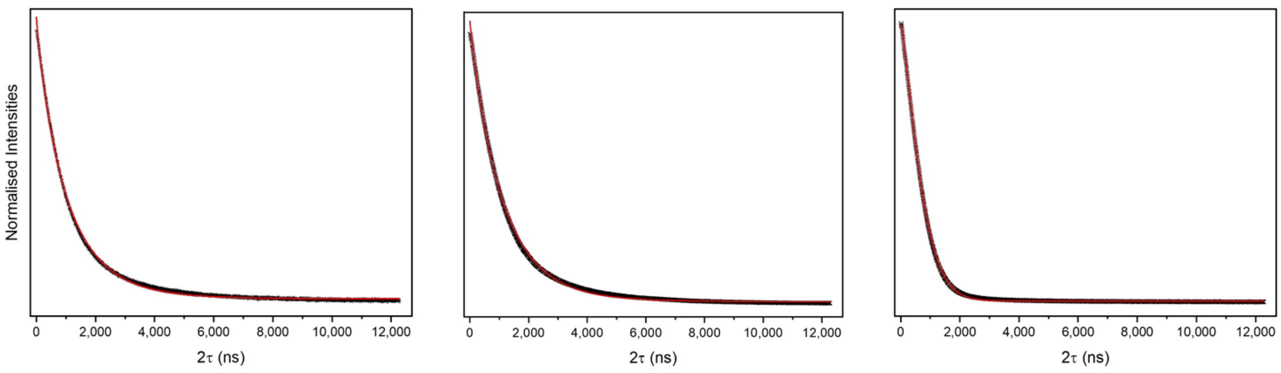

Figure S18: Phase memory times (T_m) measured of **3** at Q-Band (ca. 34 GHz) in 0.1 mM solution in dry and degassed toluene at 3 K for features at B_0 = 1045 mT (left), 1169 mT (middle) and 1291 mT (right). Experimental measurements using 20 and 40 ns π/2 and π pulses, with τ = 250 ns (black crosses) Fit to an exponential decay with the form I(2τ) = I(2τ$_0$)exp(–2τ/T_m) (solid red line).

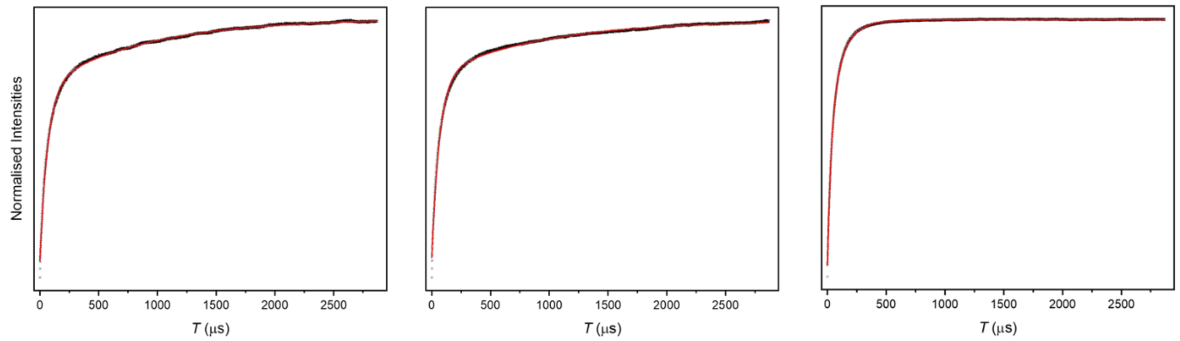

Figure S19: Spin lattice relaxation (T_1) measurements of **3** at Q-band (ca. 34 GHz), in 0.1 mM solution in dry and degassed toluene at 3 K. Measured at B_0 = 1045 mT (left), 1169 mT (middle) and 1291 mT (right), using 40 and 80 ns π/2 and π pulses, respectively, with an inversion pulse of 80 ns and T = 600 ns. Experimental trace (black crosses), and fit to an exponential decay with the form $I(T) = I(T_0)\exp(-T/T_1) + I(T_0)\exp(-T/T_{SD})$, (solid red line).

5. QUANTUM SIMULATION

5.1 Construction of Bell states

Preparation of Q_1-Q_2 maximally entangled Bell states is obtained by the following circuits (in which the two lines refer to Q_1 and Q_2 qubits):

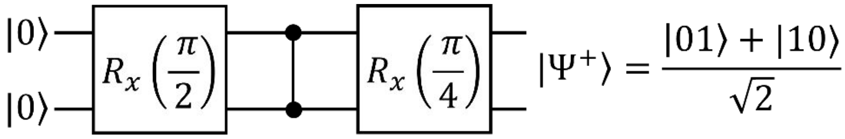

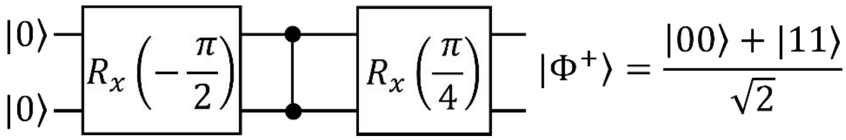

All the gates in the above construction are symmetric and hence it does not require to individually address the two qubits. In our symmetric system, simultaneous rotations of Q_1 and Q_2 are obtained with two slightly different pulses, resonant with transitions $|00\rangle \rightarrow ($

$|01\rangle + |10\rangle)/\sqrt{2}$ and $(|01\rangle + |10\rangle)/\sqrt{2} \rightarrow |11\rangle$, respectively. Hence, in the Q_1-Q_2 subspace the initial $R_x(\vartheta)$ implements the transformation:

$$R_x(\vartheta)|00\rangle = \cos^2\frac{\vartheta}{2}|00\rangle - \sin^2\frac{\vartheta}{2}|11\rangle - \frac{i}{2}\sin\vartheta(|01\rangle + |10\rangle)$$

The central controlled-Z gate is obtained by a single pulse, inducing a 2p excitation of the switch S, conditioned by the state of Q_1 Q_2.

5.2 Quantum simulation of decoherence due to system-environment interaction

We first consider a single qubit coupled to an ancilla (E) to mimic the environment. We can simulate the effect of dephasing on a generic system state $\alpha|0\rangle + \beta|1\rangle$ as follows:

- Initialize the system in a generic state, keeping E in $|0\rangle$, i.e. $(\alpha|0\rangle + \beta|1\rangle) \otimes |0\rangle$
- Implement a rotation of a tuneable angle ϑ on E:

$$R_y^{(E)}(\vartheta) \rightarrow (\alpha|0\rangle + \beta|1\rangle) \otimes (\cos\frac{\vartheta}{2}|0\rangle + \sin\frac{\vartheta}{2}|1\rangle)$$

- Implement a CNOT with the system acting as control and E as target:

$$\rightarrow \alpha\cos\frac{\vartheta}{2}|00\rangle + \beta\cos\frac{\vartheta}{2}|11\rangle + \alpha\sin\frac{\vartheta}{2}|01\rangle + \beta\cos\frac{\vartheta}{2}|10\rangle$$

The result is an evolution of E depending on the state of the system in a way controllable by changing ϑ.

- We then consider the reduced density matrix on the system state, which is given by

$$\rho_Q = \begin{pmatrix} |\alpha|^2 & \alpha\beta^*\sin\vartheta \\ \alpha^*\beta\sin\vartheta & |\beta|^2 \end{pmatrix}$$

This models pure dephasing by assuming $\sin\vartheta = e^{-t/T_2}$. ρ_Q can be obtained by full-state tomography on the system, i.e. by non-selective measurements of the expectation values of spin operators on the system (see below).

The sequence of operations above can be implemented on our molecular system by EPR pulses resonant with $E_{1,2}$ transitions. Due to the permanent coupling between Q and E, the excitation frequency of E depends on the state of Q. This allows to obtain a CNOT with a single pulse (resonant with $|1_Q 0_E\rangle \rightarrow |1_Q 1_E\rangle$), while $R_y^{(E)}(\vartheta)$ rotations require two

slightly different pulses (resonant with $|1_Q 0_E\rangle \to |1_Q 1_E\rangle$ and $|0_Q\, 0_E\rangle \to |0_Q 1_E\rangle$), to be implemented irrespectively from the state of Q.

On $|\Phi^+\rangle$ state symmetrically coupled to the bath the above sequence of operations (on both qubits) leads to the state ($\sqrt{2}$ normalization)

$$\cos^2\frac{\vartheta}{2}(|00000\rangle + |11011\rangle) + \sin^2\frac{\vartheta}{2}(|01010\rangle + |10001\rangle)$$

$$+\frac{1}{2}\sin\vartheta(|10000\rangle + |00001\rangle + |01011\rangle + |11010\rangle)$$

where we have included all five qubits (including the central ancilla). The related reduced density matrix on the Q_1-Q_2 subspace results

$$\rho_{Q_1Q_2} = \frac{1}{2}\begin{pmatrix} 1 & 0 & 0 & \sin^2\vartheta \\ 0 & 0 & 0 & 0 \\ 0 & 0 & 0 & 0 \\ \sin^2\vartheta & 0 & 0 & 1 \end{pmatrix}$$

which can again model pure dephasing between 00 and 11 components by fixing $\sin^2\vartheta = e^{-2t/T_2}$ (twice the decay rate of a single qubit, given $\Delta m = 2$ in the superposition), Initialization in $|\Psi^+\rangle$ (superposition of states with $\Delta m = 0$) leads to no dephasing.

5.3 Quantum state tomography

In order to measure the final density matrix, we can perform quantum state tomography on the Q_1-Q_2 subspace. This is achieved via a proper sequence of EPR pulses. Diagonal elements of $\rho_{Q_1Q_2}$ are obtained by measuring the probability of being in each of the three eigenstates corresponding to $|00\rangle$, $(|01\rangle + |10\rangle)/\sqrt{2}$, $|11\rangle$ irrespective from the state of $E_{1,2}$ (transitions to $(|01\rangle - |10\rangle)/\sqrt{2}$ are always forbidden due to the symmetry of the system). For instance, one could excite the central switch depending on each of the above three states of the qubits (transitions resolved by the large J coupling) and then measure the state of the switch via selective pulses.

To determine off-diagonal elements of $\rho_{Q_1Q_2}$, one needs to perform proper rotations before measurements in the ZZ basis. For instance, to measure the two-quantum coherence

$\langle 00|\rho_{QQ}|11\rangle$ one needs to compute the expectation value of an XX operator, which can be done by performing $R_y\left(\frac{\pi}{2}\right)$ on Q_1-Q_2 and then compute the difference between the resulting diagonal elements of $\rho_{Q_1Q_2}$.

6. References

S1. Whitehead, G. F. S.; Ferrando-Soria, J.; Carthy, L.; Pritchard, R. G.; Teat, S. J.; Timco, G. A.; Winpenny, R.E.P. Synthesis and reactions of N-heterocycle functionalised variants of heterometallic {Cr_7Ni} rings. *Dalton Trans*. **2016**, 45, 1638-1647.

S2. (a) Sheldrick, G. M. *SADABS*, empirical absorption correction program based upon the method of Blessing. (b) Krause, L.; Herbst-Irmer, R.; Sheldrick, G. M.; Stalke, D. An empirical correction for absorption anisotropy *J. Appl. Cryst.* **2015**, *48*. (c) Blessing, R. H. An empirical correction for absorption anisotropy, *Acta Crystallogr.* **1995**, *A51*, 33-38.

S3. (a) Sheldrick. G. M. Crystal structure refinement with SHELXL, *Acta Crystallogr.*, 2015, *C71*, 3-8; (b) Dolomanov, O. V.; Bourhis, L. J.; Gildea, R. J.; Howard, J. A. K.; Puschmann, H. OLEX2: a complete structure solution, refinement and analysis program. *J. Appl. Cryst.*, **2009,** *42*, 339–341

S4. Stoll, S.; Schweiger. A. EasySpin, a comprehensive software package for spectral simulation and analysis in EPR. *J. Magn. Reson*. **178**, 42-55 (2006).
